# Remote Ischemic Post‐Conditioning Preserves the Neurovascular Unit by Brain‐Derived Gas6 That Drives Monocyte‐Derived Macrophage Reprogramming via Axl and Vimentin after SAH

**DOI:** 10.1002/advs.77946

**Published:** 2026-09-27

**Authors:** Yajun Zhu, Zichao Huang, Xi Rong, Xingwei Lei, Fuming Liang, Xiaoguo Li, Jiru Zhou, Yu Wang, Yifan Huang, Shuang Tang, Jianjun Zhong, Jin Yan, Hongji Deng, Chun Zeng, Zongduo Guo

**Affiliations:** ^1^ Department of Neurosurgery The First Affiliated Hospital of Chongqing Medical University Chongqing China; ^2^ Department of Cerebrovascular Diseases Suining Central Hospital Sichuan China; ^3^ West China Hospital Sichuan University Sichuan China

**Keywords:** Axl, Gas6, monocyte‐derived macrophages, neurovascular unit, remote ischemic post‐conditioning, subarachnoid hemorrhage, Vimentin

## Abstract

Subarachnoid hemorrhage (SAH) causes long‐term cognitive dysfunction due to early brain injury, but effective therapies are limited. Remote ischemic post‐conditioning (RIPostC) confers neuroprotection, but its mechanisms remain incompletely understood. Here we show that RIPostC preserves the neurovascular unit by modulating monocyte‐derived macrophages (MDMs) via the Gas6/Axl/Vimentin axis. In 405 SAH patients, elevated cerebrospinal fluid leukocyte counts correlate positively with Hunt‐Hess and 3‐month mRS scores. In a rat SAH model, RIPostC suppresses periventricular CCR2^+^ cell infiltration, reduces MDMs, shifts their polarization from M1‐like to M2‐like, preserves blood‐brain barrier integrity, and improves cognitive function. Mechanistically, RIPostC downregulates Vimentin. However, the Vimentin inhibitor Withaferin A mimics anti‐infiltration but does not promote M2‐like polarization. RIPostC upregulates neuronal Gas6, which engages MDM Axl. Besides, Gas6 knockdown abolishes RIPostC's protective effects. Additionally, RIPostC selectively expands a reparative Stab1^+^Clec10a^+^ MDM subset. In human THP‐1 cells, recombinant Gas6 suppresses Vimentin and induces M2‐like polarization via Axl, and the Axl inhibitor R428 blocks this effect. Thus, RIPostC enhances neuronal Gas6 to activate MDM Axl and downregulate Vimentin, limiting infiltration and driving reparative polarization, which preserves neurovascular unit integrity and improves cognition after SAH.

## Graphical abstract

Subarachnoid hemorrhage (SAH) triggers monocyte‐derived macrophage (MDM) infiltration and M1‐like polarization via Vimentin upregulation, leading to neurovascular unit disruption and cognitive dysfunction. Remote ischemic postconditioning (RIPostC) upregulates neuronal Gas6, which activates Axl on MDMs to downregulate Vimentin and drive reparative M2‐like (Stab1^+^Clec10a^+^) polarization. This dual mechanism limits MDM infiltration and promotes neurovascular unit repair, ultimately improving cognition after SAH.

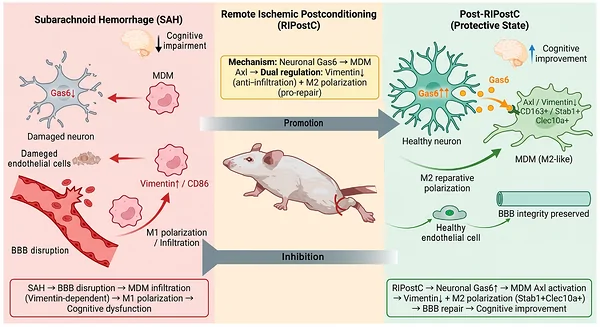

## 1 Introduction

Aneurysmal subarachnoid hemorrhage (SAH) remains a devastating cerebrovascular emergency with high mortality and long‐term disability among survivors [1]. Although advances in microsurgical clipping and endovascular coiling have reduced acute case fatality rates, a substantial proportion of patients suffer from persistent cognitive dysfunction, severely compromising their quality of life [2, 3]. Accumulating evidence implicates early brain injury (EBI), characterized by blood‐brain barrier (BBB) disruption, neuroinflammation, and neuronal apoptosis, as the primary pathological substrate underlying these poor cognitive outcomes [4]. EBI further initiates a cascade of secondary pathological events, including microcirculatory dysfunction, cortical spreading depolarization, and neuroinflammation, which may collectively predispose to delayed cerebral ischemia (DCI), another contributor to morbidity and an independent predictor of neuropsychological deficits after SAH [5, 6, 7]. However, the dynamic cellular and molecular processes that govern EBI progression are not well understood, which hinders the development of targeted therapies.

Monocyte‐derived macrophages (MDMs) represent a major population of peripheral immune cells that infiltrate the brain parenchyma following SAH‐induced BBB disruption, establishing a close spatiotemporal association with the neurovascular unit (NVU) [8, 9]. MDMs exhibit a dualistic role in the injured brain [10, 11]. On the one hand, they can promote neuroinflammation by polarizing towards a pro‐inflammatory phenotype (often termed M1‐like), releasing cytokines that disrupt BBB integrity. On the other hand, they can facilitate tissue repair by a reparative phenotype (commonly termed M2‐like). Although we recognize that the M1/M2 dichotomy is an oversimplification of the complex phenotypic spectrum observed in vivo, we employ these operational terms here to facilitate comparison with prior literature. Critically, the balance between these opposing fates influences the crosstalk among MDMs, endothelial cells (ECs), and neurons, ultimately determining the extent of NVU restoration. Its integrity is essential for normal cognitive function [12]. Therefore, therapeutic strategies that simultaneously inhibit MDM infiltration and promote their polarization towards a reparative phenotype hold significant promise for preserving NVU integrity and improving cognitive function in SAH.

Remote ischemic postconditioning (RIPostC), a non‐invasive physical therapy involving brief cycles of limb ischemia‐reperfusion, has emerged as a promising neuroprotective strategy [13]. Preclinical studies have consistently demonstrated the neuroprotective effects of RIPostC in various stroke models [14, 15, 16]. Besides, our group has previously shown that RIPostC attenuates peripheral immune cell infiltration, inhibits neuroinflammation, and ameliorates cognitive dysfunction in SAH models [14, 15]. However, the clinical evidence remains inconclusive and controversial, especially in hemorrhagic stroke [17, 18, 19]. The precise mechanisms by which RIPostC governs the behavior of MDMs still remain elusive. Specifically, the mechanism by which it simultaneously restricts their infiltration to brain and promotes their phenotypic switch are enigmatic. Furthermore, the critical molecular mediators regulating the subsequent MDM‐endothelial‐neuronal crosstalk for NVU repair are yet to be identified.

To address these gaps, we propose that the dynamic regulation of MDM infiltration and polarization within the NVU may represent a critical nexus linking long‐term cognitive impairment after SAH. We hypothesize that RIPostC may exert its neuroprotective effects by concurrently restricting MDM trafficking across the damaged BBB and steering their phenotypic switch, thereby preserving NVU integrity. However, the specific molecular machinery enabling this dual control remains to be elucidated. Notably, RIPostC is applied as an early intervention starting immediately after SAH and repeated for several days, yet the temporal dynamics of MDMs in relation to this therapeutic window are unclear. To test whether RIPostC modulates MDM behavior during their brain infiltration period, we first examined whether cerebrospinal fluid (CSF) leukocyte counts, which may reflect MDM dynamics, correlate with disease severity and outcome in a large SAH patient cohort, and whether their peak aligns with the RIPostC intervention window. Subsequently, we employed a rat SAH model to investigate the impact of RIPostC on MDM infiltration, polarization, and the underlying mechanisms, integrating single‐cell transcriptomic profiling, flow cytometry, pharmacological interventions, and in vivo gene knockdown to delineate the key signaling pathways through which RIPostC modulates MDM‐endothelial‐neuronal crosstalk.

## 2 Results

### 2.1 CSF Leukocyte Counts Peak Within the RIPostC Therapeutic Window and Correlate With SAH Severity and Poor Prognosis

**FIGURE 1:**
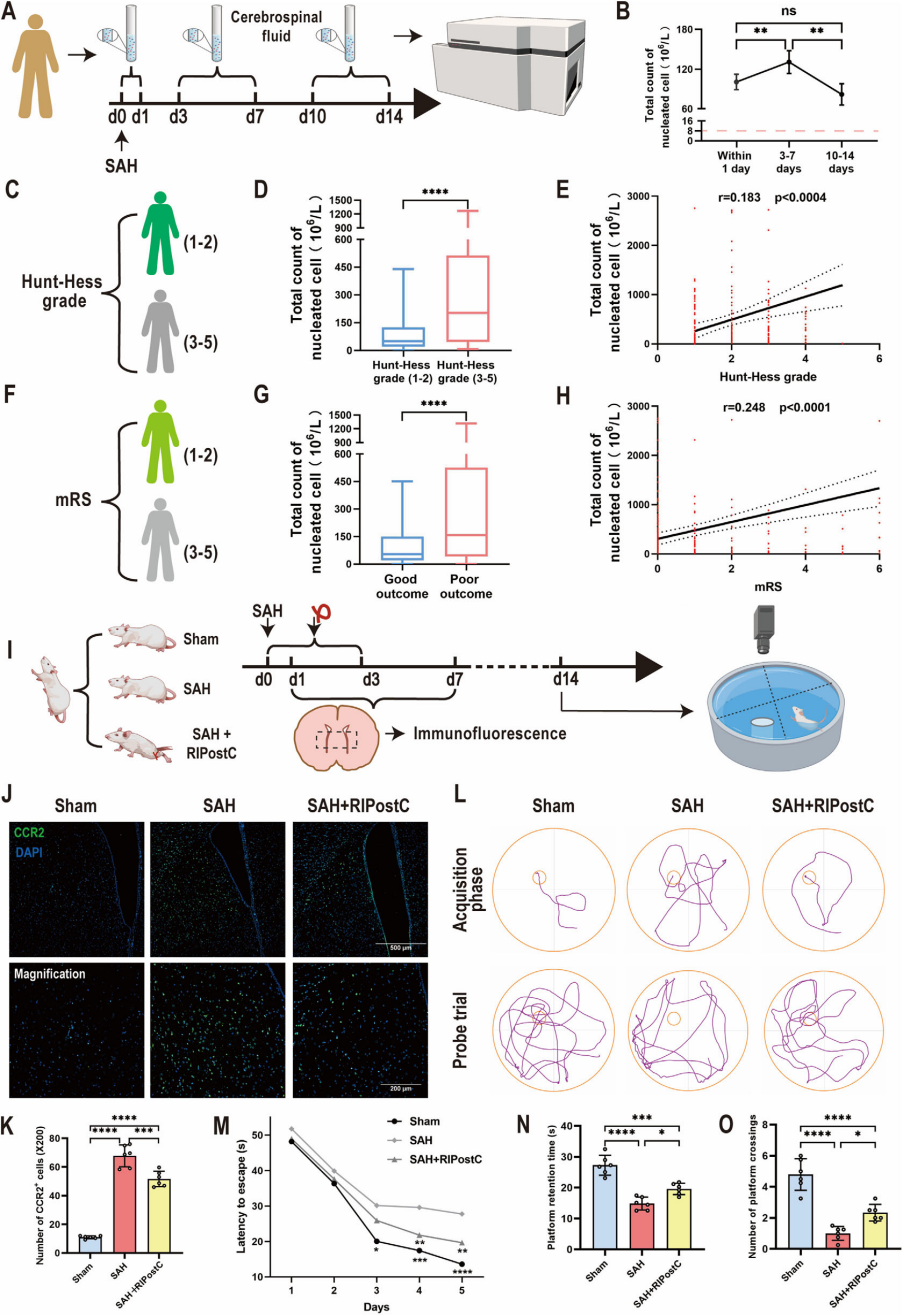
CSF leukocyte counts correlate with SAH severity and prognosis, and RIPostC reduces periventricular CCR2^+^ cell infiltration and improves cognitive function in SAH rats. (A) Schematic diagram of the clinical study workflow. (B) Dynamic changes in CSF leukocyte counts at three predefined time windows: within 24 h, days 3–7, and days 10–14 after SAH onset (*n* = 405). Dotted line indicates the upper limit of the normal reference range (8 × 10^6^ cells/L). (C–E) CSF leukocyte counts during days 3–7 stratified by Hunt‑Hess grade: mild (grades 1–2, *n* = 219) versus severe (grades 3–4, *n* = 186). (F) Grouping Diagram. (G) Comparison between groups. (H) Correlation analysis with Hunt‑Hess scores (*r* = 0.183, *p* = 0.004). (F–H) CSF leukocyte counts during days 3–7 stratified by 3‑month modified Rankin Scale (mRS) scores: good outcome (mRS 0–2, *n* = 234) versus poor outcome (mRS 3–6, *n* = 171). (C) Grouping Diagram. (D) Comparison of leukocyte counts between groups. (E) Correlation analysis between leukocyte counts and mRS scores (*r* = 0.248, p < 0.001). (I) Experimental schematic of RIPostC intervention and behavioral/cellular assessments in rats. (J, K) Representative immunofluorescence images (J) and semi‐quantitative analysis (K) of CCR2^+^ cells (green) in the periventricular region of Sham, SAH, and SAH + RIPostC groups at day 3 after SAH. Nuclei were counterstained with DAPI (blue). Scale bar, 500 µm and 200 µm. *n* = 6 per group. (L–O) Morris water maze test performed 14 days after SAH. (L) Escape latency during the 5‐day acquisition phase. (M) Representative swim trajectories during the acquisition phase (day 5) and probe trial (day 6). (N) Time spent in the target quadrant during the probe trial. (O) Number of platform crossings. *n* = 6 per group. Data are presented as mean ± SD. *p < 0.05, **p < 0.01, ***p < 0.001, ****p < 0.0001, ns: not significant.

To determine whether peripheral immune cell dynamics are temporally aligned with the therapeutic window of RIPostC, we analyzed CSF leukocyte counts in 405 SAH patients at three predefined time windows: within 24 h, days 3–7, and days 10–14 after onset (Figure 1A). Compared with normal reference values (0–8 × 10^6^ cells/L), CSF leukocyte counts were markedly elevated within the first 24 h, and continued to increase and peaked during days 3–7. It showed a declining trend but remained above normal levels at days 10–14 (Figure 1B). Notably, the days 3–7 window corresponds precisely to the period when RIPostC is actively administered in our subsequent animal experiments.

To investigate the association between CSF leukocyte counts and disease severity, patients were stratified according to the Hunt‐Hess scale (mild: grades 1–2, severe: grades 3–4) and the modified Rankin Scale (mRS) at 3 months (good: mRS 0–2, poor: mRS 3–6). Baseline characteristics of the stratified cohorts are summarized in Tables S1 and S2. We found that patients with severe SAH exhibited significantly higher CSF leukocyte counts during days 3–7 compared with those with mild SAH (Figure 1C,D). Similarly, patients with poor outcomes demonstrated significantly elevated CSF leukocyte counts during the same time window (Figure 1F,G). Furthermore, correlation analyses revealed that CSF leukocyte counts were positively correlated with Hunt‐Hess scores (*r* = 0.183, *p* = 0.004) and mRS scores (*r* = 0.248, *p* < 0.001) (Figure 1E,H). This suggest that elevated CSF leukocyte counts are associated with greater disease severity and worse functional outcomes in SAH patients.

### 2.2 RIPostC Suppresses Periventricular CCR2^+^ Cell Infiltration and Ameliorates Cognitive Dysfunction in SAH Rats

Given that CSF leukocyte counts peaked during the RIPostC intervention window in patients, we next investigated whether RIPostC directly affects the infiltration of immune cells into the brain parenchyma in a rat SAH model. We first examined CCR2^+^ cells, as CCR2 is a key chemokine receptor mediating the trafficking of of peripheral immune cells (Figure 1I). Immunofluorescence staining revealed that CCR2^+^ cells began to infiltrate the periventricular region within 24 h after SAH, peaked at day 3, and gradually declined thereafter (Figure S1). Notably, RIPostC treatment significantly reduced the number of CCR2^+^ cells in the periventricular region compared with the SAH group (Figure 1J,K).

We next assessed the effect of RIPostC on cognitive function using the Morris water maze test (Figure 1L–O). During the five‐day training phase, rats in the SAH group exhibited prolonged escape latency compared with the Sham group, indicating impaired learning ability. In contrast, RIPostC treatment significantly shortened the escape latency starting from day 4 of training (Figure 1M). In the probe trial on day 6, SAH rats showed fewer platform crossings and reduced time spent in the target quadrant compared with Sham rats. RIPostC treatment markedly increased both the number of platform crossings and the time spent in the target quadrant (Figure 1N,O).

### 2.3 Single‐Cell Transcriptomic Profiling Identifies MDMs as a Key Target of RIPostC

**FIGURE 2:**
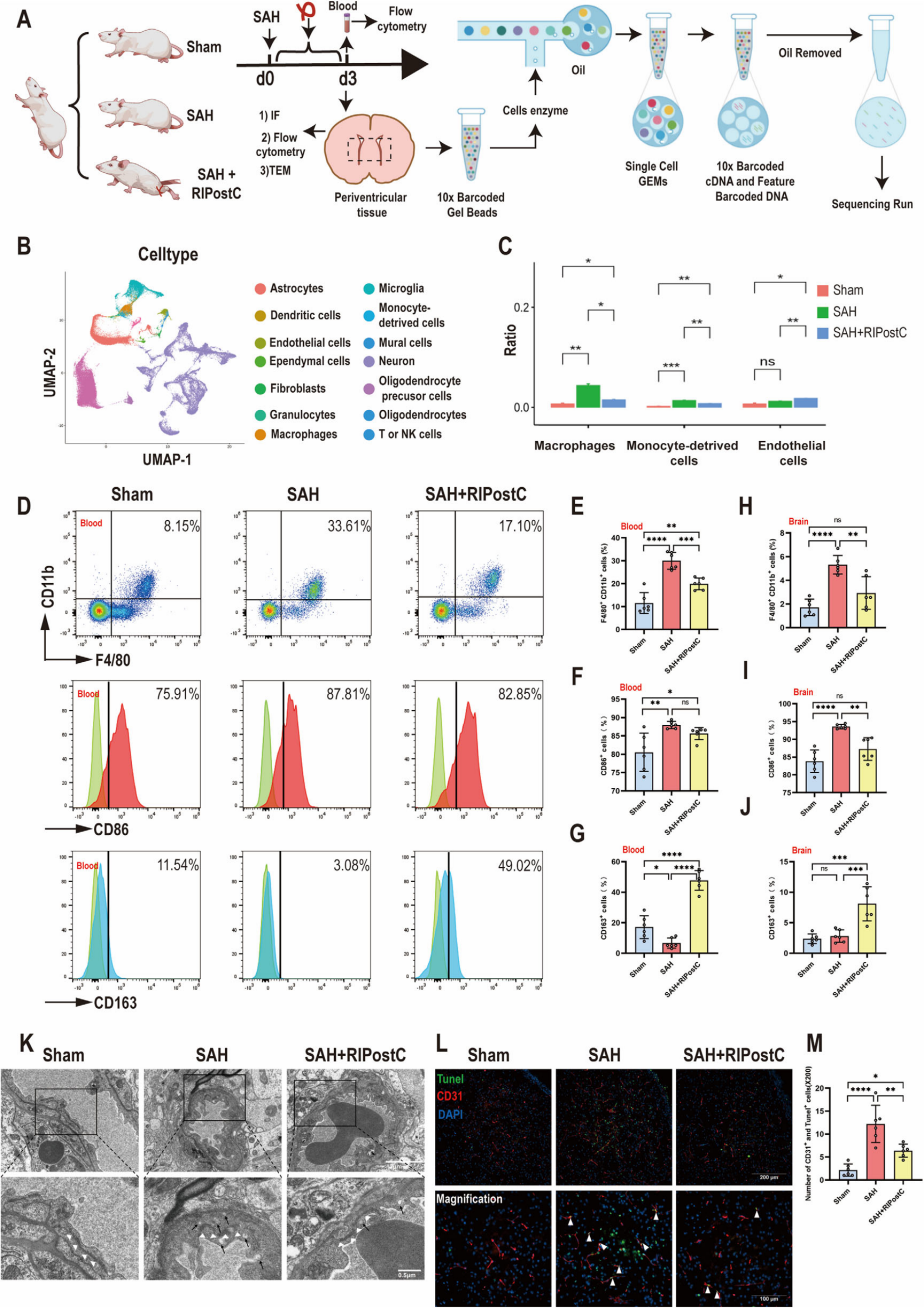
Single‑cell transcriptomic profiling identifies MDMs as a key target of RIPostC, and RIPostC suppresses MDM infiltration, promotes M2‐like polarization, and preserves BBB integrity after SAH. (A–C) scRNA‑seq of periventricular tissues from Sham, SAH, and SAH + RIPostC groups. (A) Experimental workflow. (B) UMAP plot shows 14 distinct cell populations. (C) Relative proportion of three cell types (macrophages, monocyte‐derived cell, and endothelial cell) across the three groups. (D–G) Flow cytometric analysis of peripheral blood mononuclear cells. (D) Representative gating strategy for F4/80^+^CD11b^+^ MDMs (top), CD86^+^ cells (middle), and CD163^+^ cells (bottom) in peripheral blood. (E) Quantification of percentage of F4/80^+^CD11b^+^ MDMs. (F) Quantification of percentage of CD86^+^ cells among MDMs. (G) Quantification of percentage of CD163^+^ cells among MDMs. *n* = 6 per group. (H–J) Flow cytometric analysis of periventricular brain tissues. (H) Quantification of percentage of F4/80^+^CD11b^+^ MDMs. (I) Quantification of percentage of CD86^+^ MDMs. (J) Quantification of percentage of CD163^+^ MDMs. *n* = 6 per group. (K) Transmission electron microscopy images show the ultrastructure of the BBB in each group. White arrowheads indicate tight junction, and black arrowheads indicate endothelial vesicles. Scale bar, 0.5 µm. (L, M) Immunofluorescence staining for endothelial apoptosis. (L) Representative images of CD31 (red), TUNEL (green), and DAPI (blue) in the periventricular region. White arrowheads indicate CD31^+^TUNEL^+^ double‑positive cells. Scale bar, 100 and 200 µm. (M) Semi‐quantification of CD31^+^TUNEL^+^ apoptotic endothelial cells per group. *n* = 6 per group. Data are presented as mean ± SD. *p < 0.05, **p < 0.01, ***p < 0.001, ****p < 0.0001, ns: not significant.

To delineate the cellular landscape of the periventricular region after SAH and the impact of RIPostC, we performed single‐cell RNA sequencing (scRNA‐seq) on periventricular tissues from three groups including Sham, SAH, and SAH+RIPostC groups (Figure 2A). Unsupervised clustering resolved 14 distinct cell populations, including neurons, microglia, astrocytes, oligodendrocytes, oligodendrocyte precursor cells (OPCs), dendritic cells (DCs), fibroblasts, ECs, mural cells, granulocytes, ependymal cells, macrophages, monocyte‐derived cells, and T/NK cells (Figure 2B).

As for Immune cell dynamics, compared with the Sham group, SAH rats exhibited a marked increase in the proportions of macrophages, monocyte‐derived cells, T/NK cells, DCs, and granulocytes (Figure S2). Strikingly, RIPostC treatment suppressed the increasing of all these immune populations, with macrophages showing the most pronounced reduction (Figure 2C). It suggested that macrophages were the most responsive immune subset, we focused subsequent analyses on this cell type. In addition, RIPostC treatment significantly increased the proportion of ECs compared with the SAH and Sham groups (Figure 2C). OPCs were substantially reduced after SAH, but this decrease was not rescued by RIPostC (Figure S2). Mural cells displayed a mild elevation after SAH, which was further augmented by RIPostC (Figure S2). No statistically significant changes were observed in oligodendrocytes, astrocytes, microglia, or neurons across groups (Figure S2).

### 2.4 RIPostC Suppresses MDM Infiltration, Promotes M2‐Like Polarization, and Preserves BBB Integrity

Given that scRNA‐seq identified macrophages as the most responsive immune subset and revealed a marked increasing of ECs following RIPostC treatment, we next investigated macrophage dynamics and BBB integrity using flow cytometry and functional assays. We first analyzed peripheral blood samples (Figure 2D–G). Compared with Sham rats, SAH rats exhibited a significant increase in the proportion of F4/80^+^CD11b^+^ MDMs, which predominantly expressed the M1‐like marker CD86. RIPostC treatment not only reduced the total number of F4/80^+^CD11b^+^ cells and CD86^+^ M1 cells but also increased the proportion of CD163^+^ M2‐like macrophages. Besides, similar trends were observed in brain tissue (Figure 2H–J). SAH induced a marked infiltration of F4/80^+^CD11b^+^ MDMs into the periventricular region, with a predominance of the CD86^+^ M1‐like phenotype. RIPostC significantly decreased the number of infiltrating macrophages and simultaneously promoted their polarization toward the CD163^+^ M2‐like phenotype.

We next evaluated the integrity of the BBB. Immunofluorescence staining for TUNEL revealed that SAH markedly increased the number of apoptotic ECs (CD31^+^TUNEL^+^), whereas RIPostC treatment substantially reduced endothelial apoptosis (Figure 2L,M). Transmission electron microscopy further demonstrated that SAH disrupted the ultrastructure of the BBB, characterized by discontinuous tight junctions and an increased number of endothelial vesicles. RIPostC treatment restored the continuity and integrity of tight junctions and reduced vesicle formation (Figure 2K). What's more, similar trends in neuronal apoptosis were observed and are presented in Supplementary Figure S3. Collectively, these results indicate that RIPostC suppresses MDM infiltration, shifts their polarization toward a reparative M2‐like phenotype, and protects BBB integrity after SAH.

### 2.5 RIPostC Inhibits MDM Infiltration via Vimentin

**FIGURE 3:**
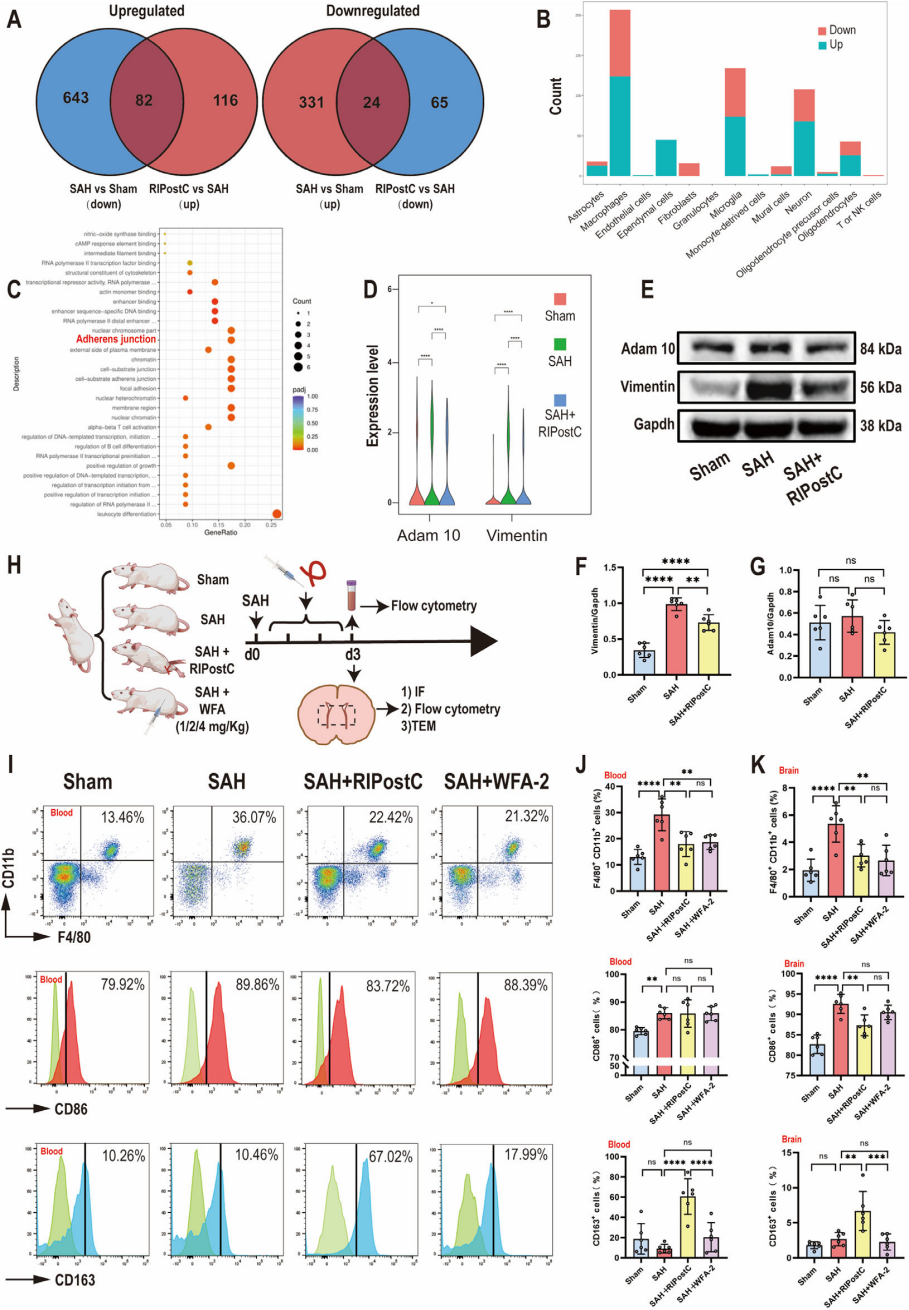
RIPostC downregulates Vimentin in macrophages, and pharmacological inhibition of Vimentin with WFA mimics the anti‑infiltration but not the pro‑polarization effects of RIPostC. (A, B) Differential gene expression analysis from scRNA‑seq data. (A) Pie chart shows the numbers of downregulated (*n* = 82) and upregulated (*n* = 24) genes. (B) Illustrating the distribution of upregulated and downregulated genes across different cell types. (C, D) Gene Ontology (GO) enrichment analysis of the downregulated genes. (C) Bubble plot of significantly enriched GO terms, with “adherens junction” highlighted. (D) Violin plot shows the involvement of Vimentin and Adam10 within the adherens junction term. (E–G) Western blot analysis of Vimentin and Adam10 protein expression in periventricular tissues. (E) Representative blots for Vimentin, Adam10, and GAPDH. (F) Quantitative analysis of Vimentin protein levels. (G) Quantitative analysis of Adam10 protein levels. *n* = 6 per group. (H) Experimental schematic illustrates the addition of the Vimentin inhibitor WFA treatment group in SAH rats. (I–K) Flow cytometric analysis of MDM infiltration and polarization after WFA treatment. (I) Representative flow cytometry plots of F4/80^+^CD11b^+^ MDMs (top), CD86^+^ cells (middle), and CD163^+^ cells (bottom). (J) Quantification of percentage of F4/80^+^CD11b^+^, CD86^+^, and CD163^+^ cells in peripheral blood. (K) Quantification of percentage of F4/80^+^CD11b^+^, CD86^+^, and CD163^+^ cells in the periventricular region. *n* = 6 per group. Data are presented as mean ± SD. *p < 0.05, **p < 0.01, ***p < 0.001, ****p < 0.0001, ns: not significant.

To elucidate the molecular mechanism by which RIPostC regulates MDM infiltration, we performed differential gene expression (DEG) and GO enrichment analyses on the scRNA‐seq data. Genes that were upregulated in SAH but downregulated by RIPostC were defined as “downregulated”, whereas those with the opposite pattern were defined as “upregulated”. In total, 82 upregulated genes and 24 downregulated genes were identified (Figure 3A). Notably, among all cell types, macrophages exhibited the highest number of regulated genes, further supporting that macrophages are a key target of RIPostC after SAH (Figure 3B). Among the downregulated functional terms, “adherens junction” was significantly enriched, containing two candidate genes: Adam10 and Vimentin (Figure 3C,D). Western blot analysis confirmed that the protein expression of Vimentin was markedly increased in the SAH group and significantly reduced by RIPostC treatment (Figure 3E,F). Although Adam10 showed a trend toward differential expression among the three groups, the changes did not reach statistical significance (Figure 3G). These results suggested that Vimentin may be a critical molecule mediating SAH‐induced MDM infiltration and is negatively regulated by RIPostC.

**FIGURE 4:**
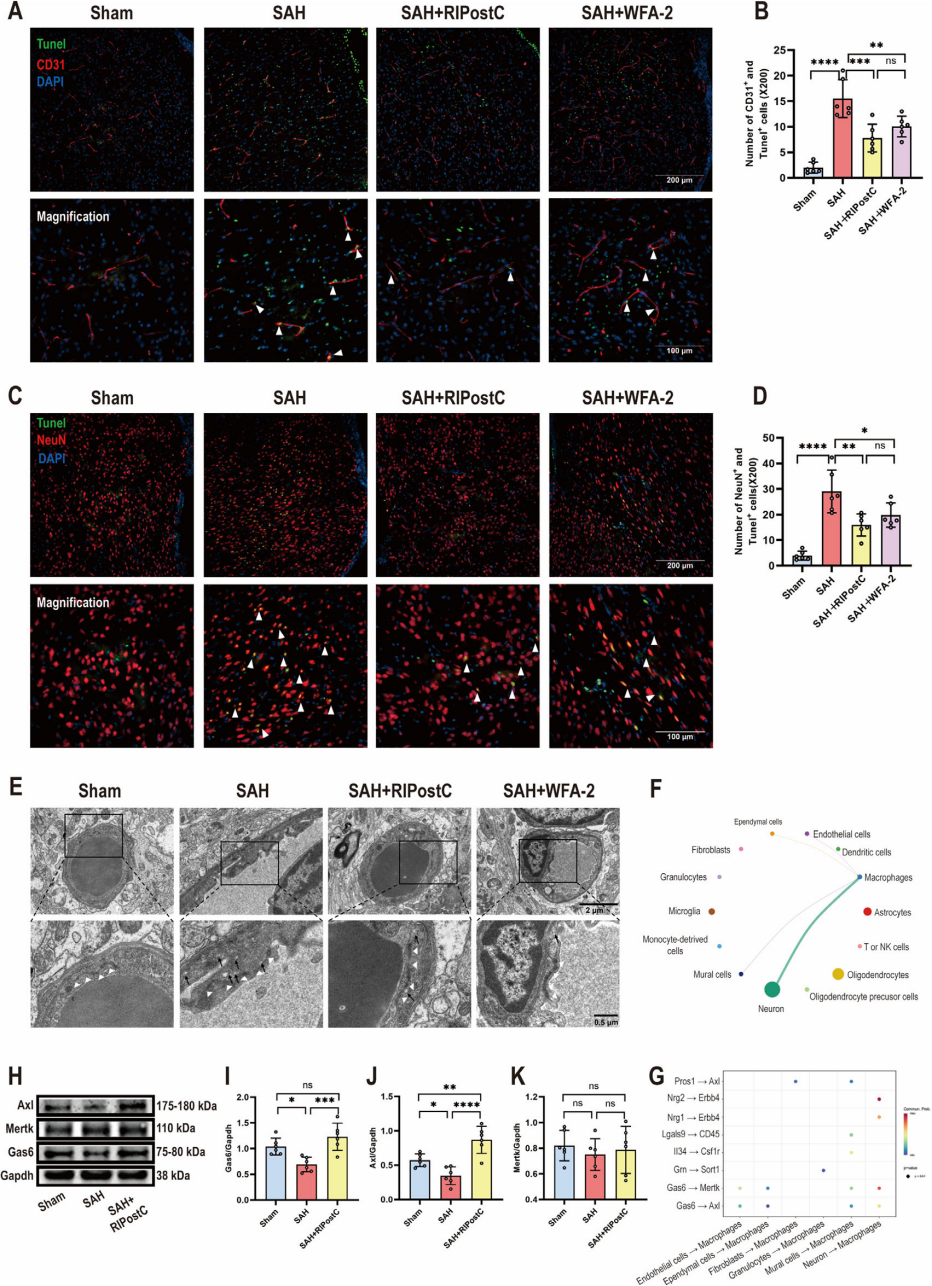
Vimentin inhibition with WFA protects BBB integrity and reduces endothelial and neuronal apoptosis, and RIPostC strengthens neuron‐MDM crosstalk via the Gas6/Axl pathway. (A) Representative immunofluorescence images of effect of WFA on endothelial apoptosis in the periventricular region. CD31 (red), TUNEL (green), and DAPI (blue). White arrowheads indicate CD31^+^TUNEL^+^ apoptotic cells. Scale bar, 100 and 200 µm. (B) Semi‐quantification of CD31^+^TUNEL^+^ cells per group. *n* = 6 per group. (C) Representative immunofluorescence images of effect of WFA on neuronal apoptosis in the periventricular region. NeuN (red), TUNEL (green), and DAPI (blue). White arrowheads indicate NeuN^+^TUNEL^+^ apoptotic cells. Scale bar, 100 and 200 µm. (D) Semi‐quantification of NeuN^+^TUNEL^+^ cells per group. *n* = 6 per group. (E) Transmission electron microscopy images show BBB ultrastructure in the Sham, SAH, SAH + RIPostC, and SAH + WFA groups. White arrowheads indicate tight junction, and black arrowheads indicate endothelial vesicles. Scale bar, 0.5 µm. (F, G) Cell–cell communication analysis based on scRNA‐seq data. (F) Circos plot showing the strength of outgoing signals from different cell types to macrophage (receiver cell). (G) Dot plot of ligand–receptor pair interactions. (H–K) Western blot analysis of Gas6, Axl, and Mertk protein expression in periventricular tissues. (H) Representative blots. (I) Quantitative analysis of Gas6. (J) Quantitative analysis of Axl. (K) Quantitative analysis of Mertk. *n* = 6 per group. Data are presented as mean ± SD. *p < 0.05, **p < 0.01, ***p < 0.001, ****p < 0.0001, ns: not significant.

To functionally validate the role of Vimentin, we administered the Vimentin inhibitor WFA (Figure 3H). As no previous study has established the optimal dose of WFA in SAH models, we performed a dose‐finding experiment. WFA was administered intraperitoneally at doses of 1, 2, and 4 mg/kg. Both 1 and 2 mg/kg suppressed Vimentin expression, with 2 mg/kg showing the greatest inhibitory effect. In contrast, 4 mg/kg unexpectedly increased Vimentin expression (Figure S4). Therefore, 2 mg/kg was selected for subsequent experiments. Flow cytometry analysis revealed that WFA treatment significantly reduced the proportion of F4/80^+^CD11b^+^ MDMs and attenuated their infiltration into the brain parenchyma compared with the SAH group (Figure 3I–K). Furthermore, WFA ameliorated BBB damage, as evidenced by reduced endothelial cell apoptosis (CD31^+^TUNEL^+^) (Figure 4A,B) and improved tight junction ultrastructure (Figure 4E). What's more, WFA similarly inhibits neuronal apoptosis after SAH (Figure 4C,D). However, unlike RIPostC, WFA did not promote the phenotypic switch of MDMs from M1‐like (CD86^+^) to M2‐like (CD163^+^) macrophage (Figure 3I–K). Based on these results, we speculate that Vimentin serves as a key downstream effector of RIPostC in regulating MDM migration, whereas the phenotypic switch of MDMs is likely governed by other upstream molecular pathways. Therefore, we preformed further investigation to identify the upstream signals that target MDMs and orchestrate their polarization.

### 2.6 RIPostC Strengthens Neuron‐MDM Crosstalk via Gas6/Axl to Repair Blood–Brain Barrier Integrity After SAH

**FIGURE 5:**
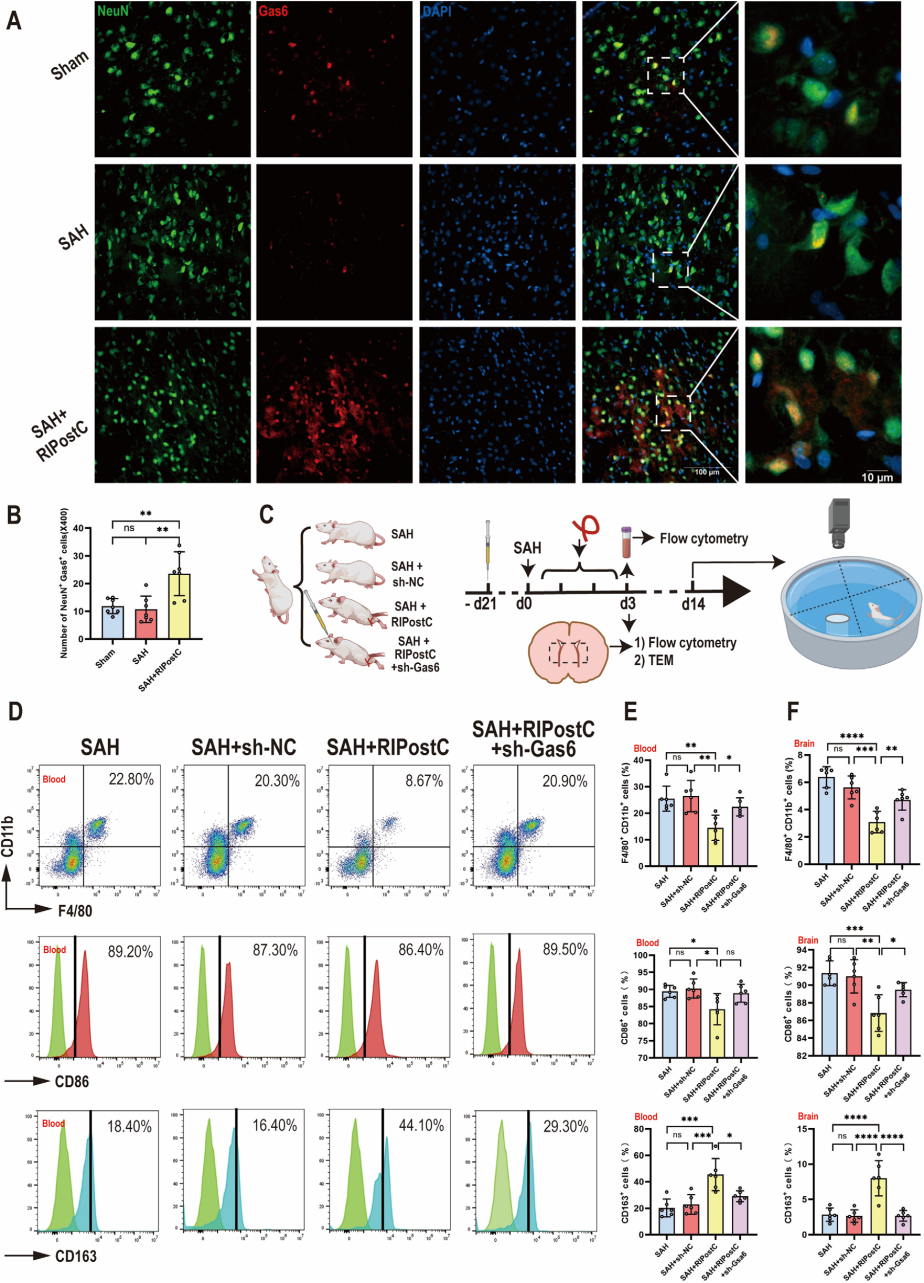
Neuronal Gas6 is essential for RIPostC‐induced MDM polarization after SAH. (A) Immunofluorescence double staining shows colocalization of Gas6 (green) with NeuN (red) in the periventricular region. Nuclei are counterstained with DAPI (blue). Scale bar, 10 and 100 µm. (B) Semi‐quantitative analysis of Gas6 protein expression in periventricular tissues of Sham, SAH, and SAH + RIPostC groups by immunofluorescence. *n* = 6 per group. (C) Experimental schematic illustrates the establishment of Gas6 knockdown rat model, followed by SAH modeling and RIPostC intervention. (D–F) Flow cytometric analysis of MDM infiltration and polarization in peripheral blood and periventricular brain tissue among four groups: SAH, SAH + sh‑NC, SAH + RIPostC, and SAH + RIPostC + sh‑Gas6. (D) Representative flow cytometry plots of F4/80^+^CD11b^+^ MDMs (top), CD86^+^ cells (middle), and CD163^+^ cells (bottom) in peripheral blood. (E) Quantification of the percentages of F4/80^+^CD11b^+^, CD86^+^, and CD163^+^ cells in peripheral blood. (F) Quantification of the percentages of F4/80^+^CD11b^+^, CD86^+^, and CD163^+^ cells among MDMs in periventricular brain tissue. *n* = 6 per group. Data are presented as mean ± SD. *p < 0.05, **p < 0.01, ***p < 0.001, ****p < 0.0001, ns: not significant.

To explore upstream signals that may regulate MDM phenotypic switching, we performed cell–cell communication analysis by defining macrophages as receiver cells and other brain cell types as senders. The analysis revealed that macrophages received signals from ependymal cells, ECs, mural cells, and neurons, with neurons exhibiting the most prominent influence (Figure 4F). Ligand–receptor pair analysis further indicated that Gas6/Axl and Gas6/Mertk interactions were the most broadly involved pathways (Figure 4G). To determine the source of Gas6 and the key receptor responsible for RIPostC's effects, we examined protein expression levels. Western blot analysis showed that Gas6 and Axl were significantly upregulated in the SAH+RIPostC group compared with the SAH group (Figure 4H–J). Although Mertk showed a mild increasing trend, the difference did not reach statistical significance (Figure 4K). These results suggest that RIPostC primarily may engage the Gas6/Axl signaling axis to modulate MDM polarization. In addition, immunofluorescence double staining revealed that Gas6 predominantly colocalized with the neuronal marker NeuN, and RIPostC treatment further increased Gas6 expression in neurons (Figure 5A,B). In contrast, colocalization of Gas6 with the endothelial marker CD31 was rarely observed (Figure S5). Taken together, these findings indicate that neurons communicate with MDMs via the Gas6/Axl pathway after SAH.

**FIGURE 6:**
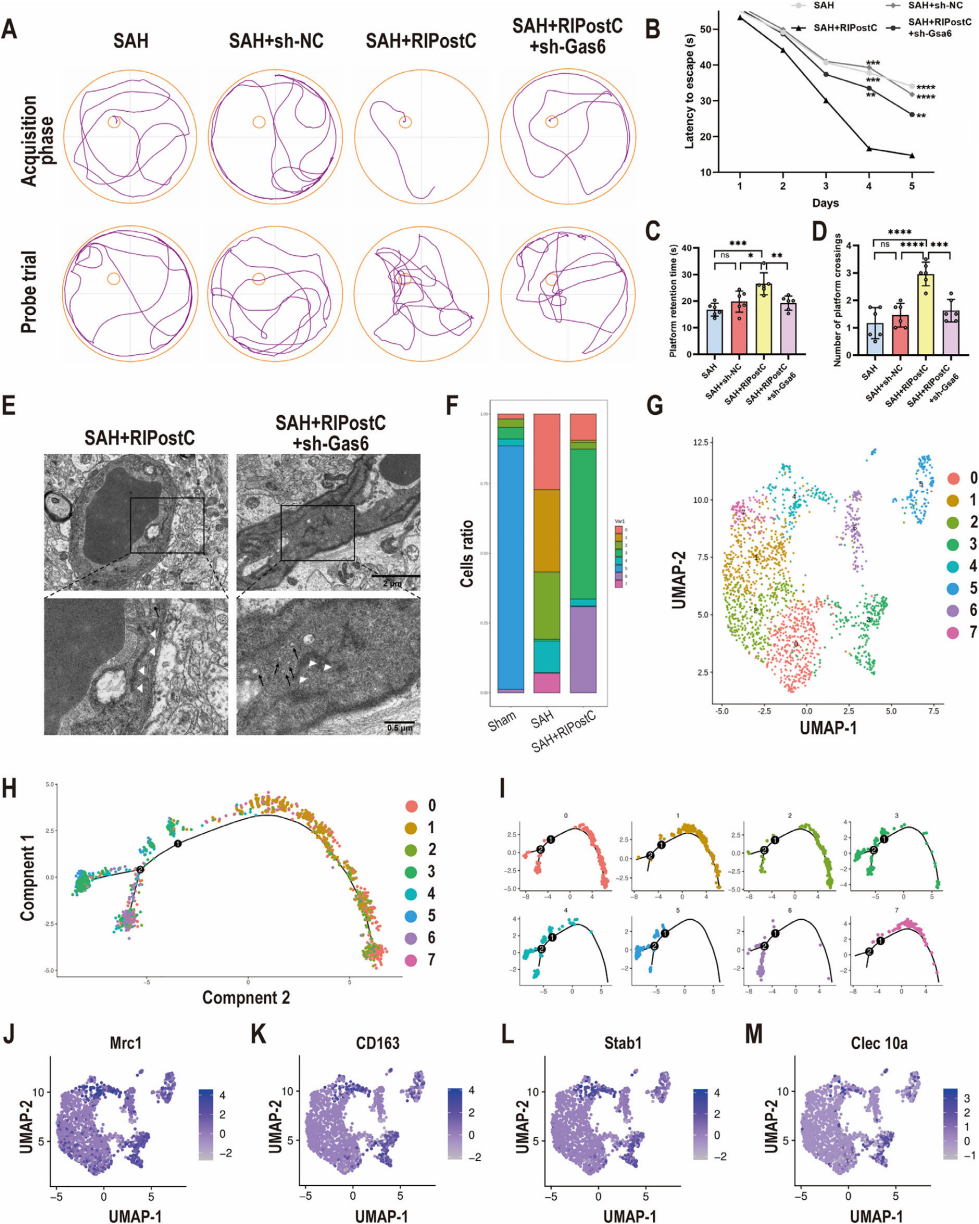
Gas6 knockdown reverses RIPostC‑induced cognitive protection and BBB integrity, and RIPostC selectively expands a Stab1^+^Clec10a^+^ reparative macrophage subset. (A–D) Morris water maze test results among four groups: SAH, SAH + sh‑NC, SAH + RIPostC, and SAH + RIPostC + sh‑Gas6. (A) Representative swim trajectories during the acquisition phase (day 5) and probe trial (day 6). (B) Escape latency during the 5‑day training period. (C) Time spent in the target quadrant during the probe trial. (D) Number of platform crossings. *n* = 6 per group. (E) Transmission electron microscopy images comparing BBB ultrastructure between SAH + RIPostC and SAH + RIPostC + sh‑Gas6 groups. White arrowheads indicate tight junction, and black arrowheads indicate endothelial vesicles. Scale bar, 0.5 µm. (F, G) Subclustering analysis of macrophages from scRNA‑seq data. (F) Bar plot shows the proportion of each macrophage subset (clusters 0–7). (G) UMAP plot visualizes the eight distinct macrophage subsets. (H, I) Pseudotime trajectory analysis of macrophage subsets. Trajectory plot shows the developmental progression from early (right) to late (left) states, with node 1 and node 2 indicating bifurcation points. (J–M) UMAP plots shows the expression levels of key genes in the reparative cluster 3. (J) Mrc1, (K) CD163, (L) Stab1, (M) Clec10a. Data are presented as mean ± SD. *p < 0.05, **p < 0.01, ***p < 0.001, ****p < 0.0001, ns: not significant.

To functionally validate this interaction, we constructed a Gas6 knockdown rat model using adenovirus‐mediated shRNA delivery (Figure 5C). Successful viral infection was confirmed by immunofluorescence detection of the virus‐encoded fluorescent reporter (GFP), and knockdown efficiency was verified by Western blot (Figure S6). Remarkably, Gas6 knockdown abolished the RIPostC‐induced reduction in MDM infiltration and the promotion of M2‐like polarization (Figure 5D–F). Moreover, Gas6 knockdown completely reversed the protective effects of RIPostC on BBB integrity and cognitive function, as evidenced by disrupted tight junction ultrastructure (Figure 6E), impaired performance in the Morris water maze test (Figure 6A–D). Therefore, these results demonstrate that the neuronal Gas6/Axl signaling axis is essential for RIPostC‐mediated MDM polarization, BBB preservation, and cognitive improvement after SAH.

### 2.7 RIPostC Promotes the Differentiation of Stab1^+^ Clec10a^+^ Reparative Macrophage Subsets After SAH

To further characterize the effect of RIPostC on macrophage polarization at the single‐cell level, we performed subclustering analysis of macrophages from the scRNA‐seq data. In SAH rats, macrophages were classified into eight distinct subsets (clusters 0—7) (Figure 6F,G). Remarkably, the proportion of cluster 3 was significantly increased in the SAH+RIPostC group compared with the SAH group (Figure 6F). Analysis of the differentially expressed genes in cluster 3 revealed high expression of Mrc1, CD163, Stab1, and Clec10a (Figure 6J–M).

Pseudotime trajectory analysis was performed to infer the developmental relationships among the eight macrophage subsets. The trajectory spanned from an early state (right side) to a late state (left side). Two critical nodes were defined including node 1, separating early from intermediate/late phases, and node 2 where the trajectory bifurcated into two distinct terminal branches (Figure 6H,I). Clusters 0, 1, 2, and 7 were predominantly located to the right of node 1, indicating that they represent early‐stage subsets. What's more, a small proportion of cells from clusters 0 and 2 also extended beyond node 2, suggesting that a minority of early‐stage cells may have the potential to transition into the terminal branches. Clusters 4, 5, and 6 were enriched to the left of node 1, suggesting they belong to intermediate or late stages. Notably, cluster 3 (the Stab1^+^ Clec10a^+^ reparative subset) was distributed across the entire trajectory but was most abundant to the left of node 1, with a marked presence at the bifurcation point and in both terminal branches (Figure 6H,I). This localization indicates that cluster 3 arises from early precursors and differentiates into a terminal reparative phenotype.

### 2.8 Gas6/Axl/Vimentin Axis Governs Human Macrophage Polarization

**FIGURE 7:**
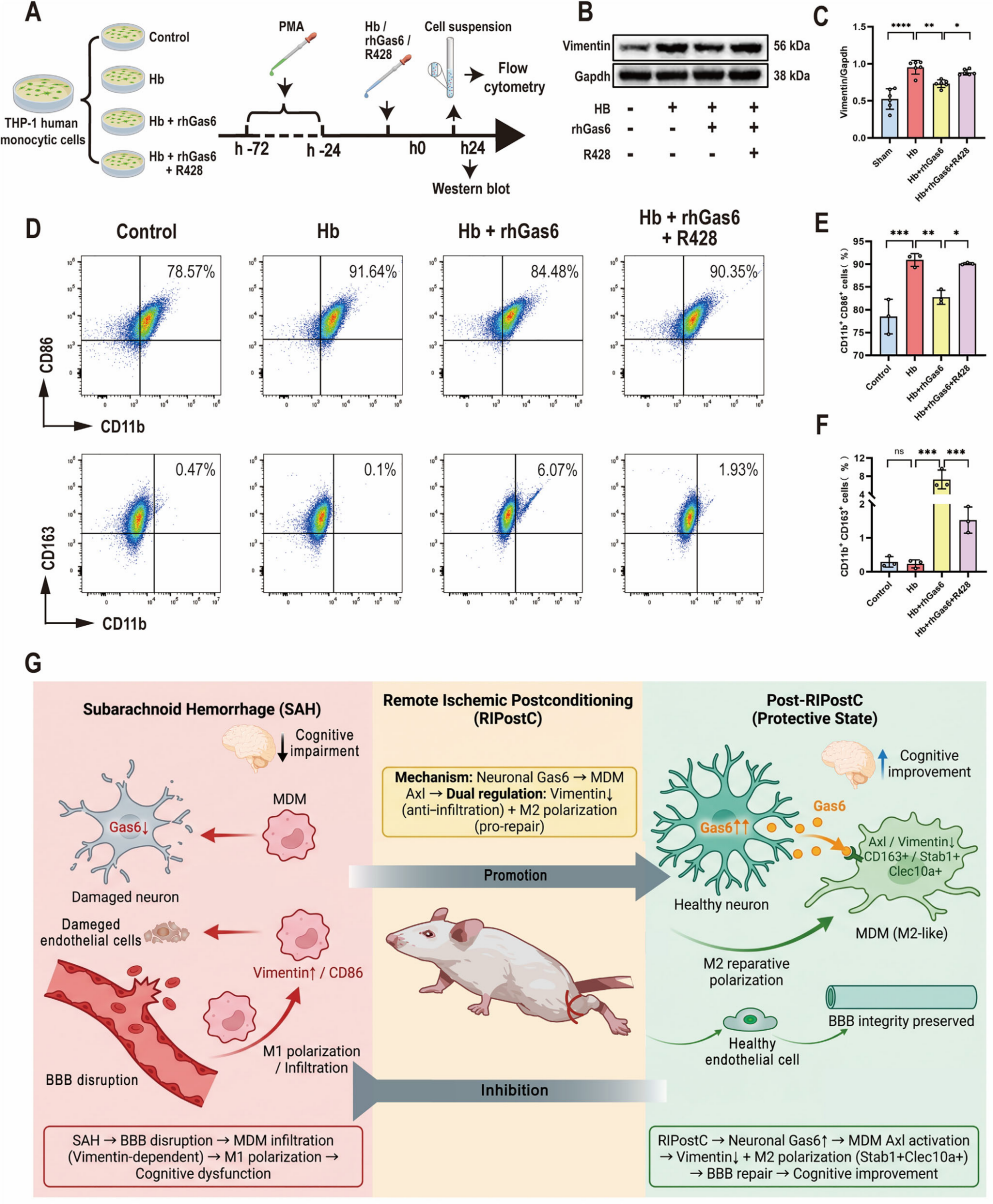
The Gas6/Axl/Vimentin axis governs THP‐1 human macrophage polarization toward an M2‐like reparative phenotype in an in vitro SAH model. (A) Experimental schematic of THP‑1 cell differentiation and treatment. (B, C) Western blot analysis of Vimentin expression in THP‑1 cell. (B)Representative blots for Vimentin and GAPDH. (C) Quantitative analysis of Vimentin expression. *n* = 6 per group. (D) Representative flow cytometry gating strategy for CD11b^+^CD86^+^ and CD11b^+^CD163^+^ macrophages among THP‑1‑derived cells. (E, F) Quantitative analysis of the percentage of CD11b^+^CD86^+^ cells (D) and CD11b^+^CD163^+^ cells (F). *n* = 3 per group. (G) Schematic diagram summarizing the proposed mechanism. Data are presented as mean ± SD. *p < 0.05, **p < 0.01, ***p < 0.001, ****p < 0.0001, ns: not significant.

To provide preclinical proof‐of‐concept supporting the clinical translation of RIPostC, we established an in vitro SAH model using human THP‐1 monocytic cells to evaluate the role of the Gas6/Axl/Vimentin axis in macrophage polarization. Four experimental groups were included. They are Control, Hb (10 µM), Hb + rhGas6 (recombinant human Gas6, 200 ng/mL), and Hb + rhGas6 + R428 (Axl inhibitor, 1 µg/mL) groups (Figure 7A).

Western blot analysis showed that Hb stimulation significantly upregulated expression of Vimentin compared with the Control group. Addition of rhGas6 markedly suppressed the Hb‐induced Vimentin upregulation. Importantly, co‐treatment with R428 reversed this suppressive effect, leading to a rebound increase in Vimentin expression (Figure 7B,C). These results indicate that Gas6/Axl signaling negatively regulates Vimentin expression in human macrophages under SAH‐like conditions.

We next examined macrophage polarization by flow cytometry. Compared with the Control group, Hb stimulation significantly increased the proportion of CD11b^+^CD86^+^ (M1‐like) cells but did not alter the percentage of CD11b^+^CD163^+^ (M2‐like) cells (Figure 7D–F). In the Hb + rhGas6 group, rhGas6 not only reduced the M1 population but also markedly elevated the M2‐like population, indicating a shift toward a reparative phenotype. However, these beneficial effects were completely abrogated by the addition of R428 in the Hb + rhGas6 + R428 group, as evidenced by a return to high M1‐like and low M2‐like levels (Figure 7D–F). Collectively, these confirm that the Gas6/Axl/Vimentin axis critically regulates macrophage polarization toward an M2‐like reparative phenotype, providing preclinical evidence that supports the clinical translation of RIPostC for SAH treatment.

## 3 Discussion

SAH frequently results in persistent cognitive dysfunction due to EBI, recognized as a major pathological driver, is characterized by BBB disruption and neuroinflammation [8, 20, 21]. MDMs critically contribute to this process, as their infiltration and polarization state decides whether inflammation is sustained or resolved [22, 23]. In this study, our clinical observation that CSF leukocytes peak during days 3–7, coinciding with the RIPostC intervention window, prompted us to investigate whether RIPostC directly modulates MDM dynamics. We further show that RIPostC simultaneously limits MDM infiltration and promotes their shift toward a reparative Stab1^+^Clec10a^+^ M2‐like phenotype through a Gas6/Axl/Vimentin axis, thereby preserving NVU integrity and improving cognitive function after SAH (Figure 7G). Notably, this study not only provides the first evidence that RIPostC acts as a molecular switch controlling both MDM migration and phenotypic reprogramming, but also identifies brain‐derived Gas6 as a critical upstream signal that engages MDM Axl to achieve these dual protective effects.

The inflammatory response after SAH plays a critical role in secondary brain injury, and mounting evidence supports the use of CSF biomarkers for prognostic assessment [24]. In our cohort of 405 SAH patients, elevated CSF leukocyte counts peaked during days 3–7 after onset. It positively correlated with both Hunt‐Hess scores and 3‐month mRS scores, reinforcing the link between intrathecal inflammation and poor clinical outcome. Our findings corroborate recent observations linking CSF leukocytosis to poor clinical course, and such temporal dynamics (days 3‑7) also align with the reported time window of DCI in previous studies [24]. However, the specific impact of RIPostC on DCI remains to be determined in future dedicated clinical investigations. In addition, compared with established molecular biomarkers such as IL‐6, TNF‐α or S100B [25], routine CSF leukocyte counting offers the advantage of being simple, cost‐effective and universally available, although its usefulness as a stand‐alone prognostic tool should be validated prospectively.

In recent years, the evidence base for RIPostC in stroke continues to expand. In ischemic stroke, the REPOST trial initially reported no significant reduction in infarct volume or improvement in mRS scores at 12 weeks [26]. But the recent SERIC‐EVT trial demonstrated that remote ischemic conditioning (RIC) improves clinical outcomes in patients treated with endovascular thrombectomy [19]. In intracerebral hemorrhage (ICH), the multi‐centre phase 3 RICH‑2 trial recently reported that RIC did not significantly improve functional outcomes in patients with supratentorial ICH, although it was safe and showed potential benefits in certain subgroups [27]. In SAH, our group has previously shown that RIPostC reduces cognitive impairment in rats and mice through modulation of Th17/Treg homeostasis and the IL‐33/ST2 axis in border‐associated macrophages [14, 15]. Extending these observations, the present study is the first to systematically dissect infiltrating MDMs as a direct target of RIPostC in SAH, and to validate the functional relevance of the Gas6/Axl/Vimentin axis in both rodent and human cells. By linking a clinically measurable inflammatory indicator (CSF leukocytosis) with a defined molecular pathway in a well‐characterized animal model, this work bridges bedside observation and bench investigation, and strengthens the translational rationale for RIPostC in SAH.

In addition, our study also demonstrates that RIPostC upregulates brain‐derived Gas6, engages Axl on MDMs, and significantly promotes M2‐like reparative polarization, including expansion of the Stab1^+^Clec10a^+^ reparative subset. We recognize that the M1/M2 classification, while widely used, does not fully capture the phenotypic and functional heterogeneity of macrophages in vivo. Nonetheless, these terms remain valuable operational descriptors for distinguishing pro‐inflammatory from reparative macrophage responses, and are employed here in this context. The Gas6/Axl pathway has consistently been shown to drive anti‐inflammatory and reparative macrophage/microglial responses across different CNS disorders. In ICH, Gas6/Axl signaling facilitated hematoma clearance and promoted microglial transition toward a protective phenotype [28]. In a mouse model of hemorrhagic transformation after ischemic stroke, recombinant Gas6 enhanced microglial M2‐like polarization via Axl activation, reducing BBB disruption and neuroinflammation [29]. Notably, the responder cells in our study are infiltrating MDMs rather than resident microglia. This cell‐type difference suggests that the ability of Gas6/Axl signaling to drive anti‐inflammatory and reparative polarization is highly conserved, regardless of whether the cells are resident or recruited. Conventional knowledge holds that Gas6 in the CNS is mainly produced by ECs, vascular smooth muscle cells, and glia [30]. Gas6 mRNA and protein are widely expressed in the cerebral cortex, hippocampus and cerebellar Purkinje neurons, underscoring its roles in development and homeostasis [31, 32]. However, whether neurons act as a major source of Gas6 and actively participate in immune regulation after SAH has been largely overlooked. We demonstrate that Gas6 predominantly colocalizes with neurons by single‑cell communication analysis and immunofluorescence, and RIPostC further increases neuronal Gas6 expression. This finding provides a new paradigm for “neuron‑macrophage” crosstalk. It suggests that neurons are not merely passive victims of inflammation but actively orchestrate immune cell behavior as upstream signaling sources. This insight broadens our understanding of neuroimmune interactions and offers a rationale for targeting neuron‑derived repair signals.

The TAM receptors (Tyro3, Axl, and Mertk) share Gas6 as a ligand but exert distinct functions depending on cell type and pathological context [33]. A previous SAH study has shown that Mertk activation regulates microglial NLRP3 inflammasome, inducing autophagy and alleviating neuroinflammation [34]. Moreover, Mertk is abundantly expressed on microglia and mediates efferocytosis of apoptotic neurons and myelin debris, which is a key repair mechanism [35, 36, 37]. In those reports, the beneficial effects of Mertk were attributed to microglia. In contrast, our study focuses on infiltrating MDMs rather than resident microglia. Compared with Axl, Mertk expression is relatively low on peripheral‑derived infiltrating macrophages, and its upregulation may depend on microglia‑specific transcriptional networks. The absence of a significant change in Mertk protein levels after SAH or RIPostC treatment likely reflects cell‑type‑specific constraints on TAM receptor expression. Therefore, RIPostC achieves dual functional regulation of MDMs through the Gas6/Axl axis. On the one hand, it downregulates Vimentin to limit MDM infiltration. On the other hand, it drives proliferation and differentiation of the Stab1^+^Clec10a^+^ reparative subset, thereby promoting M2‐like polarization. This “migration suppression + repair activation” synergy has not been previously described in SAH immunomodulation and represents a unique advantage of RIPostC neuroprotection. Others also reported that Gas6/Axl signaling is active during the recovery phase of stroke, participating in myelin debris clearance and cholesterol metabolism, indicating strong potential for long‐term repair [38, 39]. Together with our human THP‐1 validation, the Gas6/Axl/Vimentin axis may serve as a molecular biomarker for efficacy assessment and a potential therapeutic target in clinical translation of RIPostC.

Vimentin, a type III intermediate filament protein, is known to regulate fundamental cellular processes including cell migration, proliferation, and division, and has been implicated in multiple nervous system diseases [40]. Our study demonstrates that expression of Vimentin is markedly upregulated in MDMs after SAH, which can be significantly downregulated by RIPostC. Notably, pharmacological inhibition of Vimentin with WFA effectively reduced MDM infiltration into the brain parenchyma and ameliorated BBB disruption, phenocopying the anti‐infiltration effect of RIPostC. This result is consistent with the well‐established role of Vimentin in cell motility and migration. It suggests that Vimentin is a key downstream executor of RIPostC‐mediated restriction of MDM migration. However, another critical finding of our study is that WFA treatment failed to promote the phenotypic switch of MDMs from M1‐like to M2‐like, unlike RIPostC. This divergence indicates that the pro‐polarization effect of RIPostC, mediated by Gas6/Axl signaling, operates through a Vimentin‐independent pathway. This aligns with the understanding that the Gas6/Axl axis can regulate macrophage phenotype via distinct downstream signaling cascades. Previous studies have shown that Gas6/Axl signaling in macrophages acts through the PI3K‐AKT‐NF‐κB or STAT3 pathway to activate M2‐like polarization and inhibit M1‐like polarization [41, 42]. In addition, it also reported that PPARγ acts as a central transcription factor, specifying distinct M2‐like reparative subsets. It is plausible that RIPostC, via Gas6/Axl, activates STAT3 and/or PPARγ to orchestrate M2‐like polarization, independent of Vimentin. However, given the scope of the present study, we did not further dissect these downstream transcriptional mechanisms, which await rigorous investigation in future work.

Besides, Vimentin has been demonstrated to exert multiple effects in neuroinflammation and brain injury [40]. Vimentin^−^/^−^ mice exhibited reduced susceptibility to infection and a diminished inflammatory response in bacterial meningitis mice [40, 43]. Vimentin also participates in microglial activation and neurotoxicity in cerebral ischemia [43]. Recent study also highlights the dual roles of macrophages and microglia in both neuroinflammation and tissue repair after SAH [11]. Our study extends these observations by functionally separating Vimentin‐dependent migration from polarization, offering a refined view of how Vimentin contributes to SAH pathology and how RIPostC selectively exploits its regulatory functions.

In the single‑cell transcriptomic subclustering of macrophages, we identified a distinct Stab1^+^ Clec10a^+^ subset (cluster 3). It was significantly expanded by RIPostC after SAH. Both Stab1 and Clec10a genes are established markers of alternatively activated (M2‑like) macrophages with well‑documented functions in inflammation resolution and tissue repair [44, 45]. Stabilin‑1 is a scavenger receptor that mediates the clearance of apoptotic cells and promotes angiogenesis [46, 47]. Therefore, its high expression in cluster 3 suggests a role for this subset in debris clearance and vascular repair. In addition, Clec10a, also known as Mgl1, recognizes N‑acetylgalactosamine on damaged cells and has been shown to regulate macrophage polarization toward an anti‑inflammatory phenotype [48, 49]. The enrichment of these markers in cluster 3 suggests that this subset may contribute to myelin debris clearance, endothelial stabilization, and neurovascular repair after SAH. Further pseudotime trajectory analysis placed cluster 3 predominantly at the late stage of differentiation, with a strong presence at the bifurcation point and in both terminal branches. This indicates that cluster 3 represents a terminally differentiated reparative state rather than a transitional or pro‑inflammatory intermediate. This further proves that RIPostC may guide infiltrating MDMs away from sustained inflammation and toward a committed repair pathway. It's a hallmark of precise immunomodulation.

We acknowledge that the present study has not experimentally validated the functional role of the Stab1^+^Clec10a^+^ subset, such as through targeted depletion or adoptive transfer. The findings are therefore transcriptome‑based hypotheses that provide a molecular signature of RIPostC's beneficial effect. Future studies using lineage tracing, specific gene knockout, or antibody‑mediated subset blockade are required to causally link this subset to myelin repair, BBB integrity, and cognitive recovery. Nonetheless, the consistent expansion of this subset by RIPostC across individual animals and its tight association with improved outcomes make it a promising biomarker for monitoring therapeutic response and a potential target for amplifying the protective effects of RIPostC. Importantly, the identification of the Stab1^+^Clec10a^+^ subset not only supports the concept of reparative macrophage polarization beyond the traditional M2 marker (CD163) but also provides a more refined molecular signature of RIPostC‐induced protective immunomodulation, moving beyond the limitations of the classical M1/M2 dichotomy.

As for anesthetic consideration, we acknowledge that several anesthetics, including propofol, isoflurane, and dexmedetomidine, have been reported to exert neuroprotective effects in experimental stroke and SAH models [50, 51, 52]. Although pentobarbital, the anesthetic used throughout the present study, has also been investigated for its potential cerebroprotective properties, the evidence base differs substantially from that of propofol or isoflurane [53]. Barbiturates have traditionally been employed as a “last‑resort” therapy for refractory vasospasm and intractable intracranial hypertension (ICP) after SAH, with their proposed benefits attributed primarily to metabolic suppression and ICP reduction rather than to targeted modulation of specific inflammatory or oxidative stress pathways [53, 54]. However, to date, no randomized controlled trial has validated a definitive neuroprotective effect of pentobarbital in SAH. Importantly, in the present study, all experimental groups received identical pentobarbital exposure at corresponding time points, ensuring that the only variable distinguishing groups was the presence or absence of RIPostC intervention. Moreover, the complete reversal of RIPostC‑mediated protection by Gas6 knockdown (Figures 5D–F and 6A–E) provides direct genetic evidence that the observed neuroprotective phenotype is specifically dependent on the Gas6/Axl/ Vimentin axis activated by RIPostC, rather than on non‑specific anesthetic effects. Collectively, these considerations and experimental controls strongly support the conclusion that the protective effects observed in this study are specifically mediated by RIPostC‑induced molecular signaling.

In our study, RIPostC offers several advantages for clinical translation. It is non‐invasive, safe, and easily standardized. Besides, our dose‐finding study for WFA provides a reference for future clinical regimens. Lastly, the demonstration that the Gas6/Axl/Vimentin axis operates in human THP‐1 macrophages supports the translational relevance of RIPostC. We acknowledge that the optimal clinical initiation time for RIPostC in SAH patients (e.g., within 6‑‐12 h after onset) warrants future investigation, but this does not undermine the experimental rationale for early intervention in the animal model. However, several limitations should be acknowledged. First, our clinical data are from a single‐center retrospective cohort. It was also limited to total CSF leukocyte counts without differential subset analysis. Prospective and multicenter validation with dedicated immune‐phenotyping to characterize CSF leukocyte subset dynamics is warranted to definitively identify the key cell populations. Second, only male rats were used in this study. Although this design minimized the confounding effects of female sex hormones on immune responses, we recognize that SAH has a higher incidence in females and middle‐aged populations. Whether the neuroprotective effects of RIPostC and the Gas6/Axl/Vimentin axis are sex‐ or age‐dependent remains to be determined. Thirdly, whole‐brain Gas6 knockdown does not allow cell‐type‐specific conclusions. Future studies using neuron‐specific Cre lines (e.g., Cam2Cre) are needed to definitively assign the source of protective Gas6 to neurons. Fourth, although the highest WFA dose (4 mg/kg) unexpectedly increased Vimentin expression, which may reflect non‐specific effects. This observation did not interfere with our main conclusion, as the optimal inhibitory dose (2 mg/kg) was clearly identified and used throughout all functional experiments. Fifth, our definition of NVU preservation in this study primarily encompasses the functional restoration of the endothelial barrier and the reduction of neuronal injury. While we acknowledge that the responses of astrocytes and pericytes were not exhaustively interrogated, our single‑cell data provide a foundational atlas for future exploration of these cellular components. Despite these limitations, the consistency of our multi‐level data and the conserved nature of the identified pathway provide a strong rationale for further translational development of RIPostC in SAH. While our study primarily focused on EBI as the pathological driver of cognitive dysfunction after SAH, we acknowledge that DCI is another well‐established contributor to poor neurological and cognitive outcomes [5, 6, 7]. At last, whether RIPostC confers protection against DCI remains unknown and warrants future investigation. The present experimental paradigm was not designed to assess DCI, as our primary endpoints were evaluated within the acute phase of EBI.

In conclusion, this study demonstrates that RIPostC increases neuron‑derived Gas6, which engages Axl on MDMs to downregulate Vimentin, thereby simultaneously limiting MDM infiltration and driving their reparative M2‐like polarization. Mechanistically, this work advances our understanding of SAH pathophysiology by revealing a previously unrecognized neuron‑to‑MDM signaling axis that bridges neuroinflammation and vascular repair after hemorrhage. Therapeutically, the conservation of this axis in human macrophages, combined with the non‑invasive nature of RIPostC, provides a strong preclinical proof‑of‑concept for its clinical translation as a safe and feasible strategy to improve cognitive recovery in SAH patients.

## 4 Methods

### 4.1 Patients

405 patients from The First Affiliated Hospital of Chongqing Medical University were screened, which were diagnosed with SAH and admitted between January 2021 and January 2025. The inclusion criteria were as follows: (1) First‐time SAH confirmed by computed tomography angiography (CTA) or magnetic resonance angiography (MRA). (2) Admitted within 24 h after onset of symptom. (3) Age between 18 and 75 years. (4) No pre‐existing severe comorbidities leading to a loss of independent daily living capacity. Patients were excluded based on the following criteria: (1) Onset time to admission exceeding 24 h. (2) Presence of severe underlying systemic diseases such as pulmonary insufficiency, heart failure, decompensated renal failure, decompensated liver cirrhosis, etc. at onset. (3) Concomitant coagulopathy, infection or autoimmune disease. (4) Complicated with central or peripheral infection during the course of the disease. (5) History of pre‐existing neurological disorders (e.g., traumatic brain injury, neurodegenerative diseases, epilepsy) that could potentially affect baseline cognitive function or neurological status.

### 4.2 Clinical Data

Clinical data of patients were systematically extracted from electronic medical records and covered four primary domains: (1) For demographic and baseline clinical characteristics, this included gender, age, and a set of past medical history and habits: hypertension, diabetes mellitus, hyperlipidemia, history of ischemic stroke or transient ischemic attack, coronary heart disease (CHD), current smoking status, and alcohol consumption. (2) Admission severity: The initial clinical severity was assessed using the Hunt‐Hess scale (3) Outcomes: Assessed via the modified Fisher score. (4) Laboratory Investigations: Serial complete CSF samples analyses were performed at three predefined time windows (within 24 h, days 3–7, and days 10–14). The reference ranges of laboratory parameters were interpreted using the National Clinical Laboratory Operating Procedures (5th edition, ISBN: 9787117198622). The extracted parameter included leukocyte count. This study was approved by the Ethics Committee of First Affiliated Hospital of Chongqing Medical University (Approval No. ZZ2025‐887‐01). Due to the retrospective nature of the study and the use of anonymized clinical data extracted from the hospital information system, the requirement for written informed consent was waived by the ethics committee.

### 4.3 Animals

Adult male Sprague‐Dawley rats (8–10 weeks, 200–220 g) were obtained from the Animal Experiment Center of Chongqing Medical University. Male rats were used in this study to avoid the potential confounding effects of the estrous cycle on immune responses and inflammatory outcomes, which are central to our mechanistic investigations. All animals were housed under a specific pathogen‐free condition with a 12:12‐h light/dark cycle at a constant temperature (24°C ± 1°C) and humidity (50% ± 10%), with free access to food and water. Rats were randomly assigned to six experimental groups (n = 6 per group): Sham, SAH, SAH + RIPostC, SAH + WFA, SAH + sh‐NC, and SAH + RIPostC + sh‐Gas6. All experimental procedures were performed in accordance with the Guidelines for Animal Experiments of Chongqing Medical University and the ARRIVE 2.0 guidelines. The study protocol was approved by the Experimental Animal Care and Use Committee of Chongqing Medical University (IACUC, Approval No. IACUC‐CQMU‐2024‐04072).

### 4.4 Adeno‐Associated Virus‐Mediated Gas6 Knockdown

To knockdown Gas6 expression in the brain, rats received an intracerebroventricular (ICV) injection of adeno‐associated virus serotype 9 (AAV9) encoding short hairpin RNA targeting Gas6 (sh‐Gas6) or control scrambled shRNA (sh‐NC). The AAV9 vectors were constructed by (Taitool Bioscience, China) with a titer of 1.87E+13 virus genome (V.G.)/mL. Stereotaxic injections were performed 4 weeks prior to SAH induction. Rats were anesthetized with 3% sodium pentobarbital (45 mg/kg i.p.). The depth of anesthesia was confirmed by the absence of withdrawal response to hindlimb pinch. During the surgical procedure, normothermia (37.0°C ± 0.5°C) was maintained using a heating pad, and all rats were allowed to breathe spontaneously under room air. The head was fixed in a stereotaxic frame (RWD Life Science, Shenzhen, China), and a midline scalp incision was made. The skull was drilled using a specialized dentist drill, making two 1 mm holes, located above the lateral ventricles (coordinates relative to bregma: anteroposterior: −1.5 mm, mediolateral: ± 1.5 mm, and dorsoventral: + 4 mm) [55]. A 10‐µL Hamilton microsyringe (Hamilton, Reno, NV, USA) was used to inject 5 µL of AAV9 solution (containing 1 × 10^9^ V.G.) into each lateral ventricle at a rate of 0.5 µL/min. For each side, the syringe was left in place for 10 min after injection to allow diffusion and prevent backflow, then slowly withdrawn. The burr hole was sealed with bone wax, and the incision was closed.

### 4.5 Establishment of SAH Model

SAH was induced in rats via the endovascular perforation technique as previously described [14]. Briefly, after adequate anesthesia as previously mentioned in Sections 4.4, the rat was placed in the supine position, and a midline neck incision was made. The left external carotid artery (ECA) was isolated, ligated, and transected. A 4‐0 monofilament nylon suture was introduced into the internal carotid artery (ICA) through the ECA stump and gently advanced approximately 18–20 mm from the carotid bifurcation until resistance was felt. The suture was then advanced an additional 2 mm to perforate the ICA, creating a SAH. The suture was withdrawn immediately, and the ECA stump was ligated to achieve hemostasis. Sham‐operated rats underwent the same surgical procedure except for the vessel perforation step. Rats with a total SAH score ≤ 7 were excluded from subsequent analyses to ensure consistent injury severity across experimental groups as previously described [56].

### 4.6 RIPostC Intervention

RIPostC was initiated immediately after the SAH model. As previously mentioned [14], rats still maintain anesthesia. RIPostC was conducted mainly by tightening a tourniquet (8 mm) around the proximal thigh of both hind limbs simultaneously to apply pressure. Successful occlusion was confirmed by pallor of the paw and a decrease in skin temperature (> 3°C) [13]. Each cycle consisted of 10 min of ischemia followed by 10 min of reperfusion. Three cycles were performed per day for 3 consecutive days. In addition, for all subsequent RIPostC sessions, all experimental groups (including SAH and sham controls) received the same anesthetic regimen (sodium pentobarbital, 45 mg/kg, i.p.) prior to each session, ensuring identical anesthetic exposure across all groups.

### 4.7 Morris Water Maze Test

Spatial learning and memory abilities were assessed using the Morris water maze test at 14 days after SAH, as previously described [14]. The apparatus consisted of a circular pool (diameter: 150 cm, height: 60 cm) filled with water (22°C ± 1°C). A hidden platform (diameter: 12 cm) was submerged 1.5 cm below the water surface in the center of the target quadrant. The test consisted of two phases including a five‐day acquisition phase and followed by a probe trial on day 6. During the acquisition phase, each rat received four training trials per day. In each trial, the rat was placed into the pool facing the wall from one of four randomly assigned start locations and allowed to swim freely for up to 60 s to locate the hidden platform. The escape latency (time to reach the platform) was recorded for each trial. On day 6, the platform was removed, and each rat was allowed to swim freely for 60 s. The time spent in the target quadrant where the platform had been located and the number of platform crossings were recorded as measures of spatial memory retention. All swimming trajectories were recorded and analyzed using the ANY‐maze video tracking system (Stoelting, USA).

### 4.8 Single‐Cell RNA Sequencing

Periventricular tissues for scRNA‐seq were collected at day 3 after SAH, based on our observation that CCR2^+^ cell infiltration peaked at this time point (Figure S1). Single‐cell suspensions were prepared from brain tissues of rats in the Sham, SAH, and SAH+RIPostC groups, followed by single‐cell library construction using the 10X Genomics platform and sequencing on the Illumina platform. Raw data were aligned using Cell Ranger, and the three groups of data were separately quality‐controlled and filtered with Seurat, then integrated via anchor‐based batch effect correction. Principal component analysis, UMAP dimensionality reduction and clustering were performed, and cell types were annotated based on canonical marker genes. For specific cell subpopulations, differentially expressed genes were identified using the Wilcoxon rank‐sum test (|log2FC|>0.5, adjusted P<0.05), and GO and KEGG enrichment analyses were conducted using clusterProfiler to reveal transcriptomic changes induced by SAH and RIPostC intervention. In addition, a Sham+RIPostC group was not included because: (1) our preliminary data and previous reports indicate that RIPostC does not induce substantial transcriptomic changes in healthy brain tissue, and adding this group would increase animal use without addressing a specific hypothesis; and (2) to identify RIPostC‐regulated pathways specifically in the SAH context, we adopted an intersection strategy focusing on genes dysregulated in SAH vs. Sham and reversed by RIPostC (SAH+RIPostC vs. SAH). This conservative approach minimizes false‐positive signals unrelated to SAH pathology.

### 4.9 Cell Culture

THP‐1 human monocytic cells were obtained from (Procell, Wuhan, China). Cells were cultured in RPMI‐1640 medium (Servicebio, China) supplemented with 10% fetal bovine serum (FBS, Servicebio, China), 0.05 mM β‐mercaptoethanol (MCE, USA), and 1% penicillin/streptomycin (Pricella Biotechnology, China) at 37 °C in a humidified atmosphere containing 5% CO_2_. After resuscitation, cells were cultured in fresh medium in T75 flasks. Cells were maintained by replacing the culture medium every 2–3 days. When cell density reached 80%–90% confluence, cells were passaged at a ratio of 1:3. All experiments were performed using cells within 10 passages after resuscitation. To induce differentiation into macrophage‐like cells, THP‐1 cells were treated with 100 ng/mL phorbol 12‐myristate 13‐acetate (PMA, Servicebio, China) for 48 h. Following PMA treatment, cells were washed twice with phosphate‐buffered saline (PBS) and allowed to rest in PMA‐free medium for 24 h prior to further experimentation.

### 4.10 Drug Administration

#### 4.10.1 In Vivo Drug Administration

The Vimentin inhibitor Withaferin A (WFA, Selleck, USA) was administered via intraperitoneal injection [57]. WFA was dissolved in dimethyl sulfoxide (DMSO) and further diluted in sterile saline to the working concentration, with a final DMSO concentration of less than 0.1% (v/v). A preliminary dose‐finding study was conducted to determine the optimal dose, with WFA administered at doses of 1, 2, and 4 mg/kg body weight. Finally, 2 mg/kg was selected for subsequent experiments. WFA was injected three times at 24‐h intervals, with the first dose administered immediately after SAH induction. Rats in the control groups received an equivalent volume of vehicle (0.1% DMSO in saline) following the same schedule.

#### 4.10.2 In Vitro Drug Treatment

In vitro experiments, after PMA‐induced differentiation and 24 h of rest in PMA‐free medium, macrophages were treated with Oxyhemoglobin (10µM, dissolved in culture mediu, H2625, Sigma‐Aldrich, USA) for 24 h to simulate the SAH microenvironment. Following Hb treatment, cells were incubated with recombinant human Gas6 (rhGas6, 200 ng/mL, dissolved in culture mediu, MCE, USA) and/or the Axl inhibitor R428 (1 µg/mL, dissolved in DMSO and diluted in culture medium, final DMSO concentration < 0.1%, Selleck, USA) [58]. After treatment, cells were harvested for subsequent analyses. Control groups received equal volumes of the corresponding vehicles (culture medium for Hb and rhGas6, or 0.1% DMSO in culture medium for R428).

### 4.11 Flow Cytometry

#### 4.11.1 Cell Preparation

##### 4.11.1.1 Brain Tissue

Rats were euthanized, and both bilateral periventricular tissues were collected. Single‐cell suspensions were prepared using the Adult Rat Brain Tissue Gentle Dissociation Kit (RWD Life Science, China) and a single‐cell suspension preparation instrument (RWD Life Science) according to the manufacturer's instructions. The final cell pellet was resuspended in PBS for subsequent counting and staining.

##### 4.11.1.2 Peripheral Blood

Blood samples were collected via cardiac puncture into heparinized tubes. Peripheral blood mononuclear cells (PBMCs) were isolated using rat mononuclear cell separation medium (Solarbio, China) by density gradient centrifugation. Briefly, whole blood was layered onto an equal volume of separation medium and centrifuged at 1000 ×g for 20 min at room temperature. The mononuclear cell layer was carefully collected, washed twice with PBS, and resuspended in PBS for subsequent analysis.

##### 4.11.1.3 THP‐1 Cells

After treatment, THP‐1‐derived macrophages were collected by centrifugation at 300 ×g for 5 min. The supernatant was discarded, and cells were washed twice with PBS. The final cell pellet was resuspended in PBS for subsequent staining.

All cell suspensions were counted using a cell counter (Servicebio, China) to ensure a minimum of 1 × 10^6^ cells per sample prior to staining.

#### 4.11.2 Cell Staining and Flow Cytometry Analysis

After counting, cell suspensions were transferred to round‐bottom tubes. To block nonspecific binding, cells were incubated with purified anti‐CD16/32 antibody (dilution 1:100, Elabsceince, Wuhan, China) for 15 min at 4°C. Subsequently, cells were stained with the following fluorochrome‐conjugated antibodies for 45 min at 4°C in the dark: FITC anti‐F4/80 antibody (dilution 1:20, Elabsceince, China), PerCP/Cyanine5.5 anti‐CD11b antibody (dilution 1:20, Elabsceince, China), APC anti‐CD86 antibody (dilution 1:20, Elabsceince, China), and PE anti‐CD163 antibody (dilution 1:20, Thermo Fisher Scientific, USA). After staining, cells were washed twice with staining buffer by centrifugation at 300 ×g for 5 min at 4°Cand resuspended in 200–300 µL of staining buffer. Data were acquired using a CytoFLEX flow cytometer (USA) and analyzed using FlowJo software (version 10.8, USA).

### 4.12 Western Blot

Protein lysates were extracted from both bilateral periventricular tissues and cultured THP‐1 cells. The tissues or cells were then homogenized in 10 volumes of ice‐cold RIPA (Beyotime, China) supplemented with a protein inhibitor cocktail (Roche, Indianapolis, USA) to extract the protein. After centrifugation (12 000 ×g, 20 min, 4°C), supernatants were quantified via Bicinchoninic Acid (BCA) assay (Beyotime, China). Equal amounts of protein (20 µg per lane) were separated by sodium dodecyl sulfate‑polyacrylamide gel electrophoresis (SDS‑PAGE) on 10% gels and subsequently transferred to polyvinylidene fluoride membranes (Bio‑rad, USA). Then, it was sealed with the rapid sealing solution (Beyotime, China) for 20 min at room temperature, then incubated overnight at 4°C with the following primary antibodies: rabbit anti‐GAS6 (1:1000, Proteintech, China), rabbit anti‐MERTK (1:1000, Proteintech, China), rabbit anti‐AXL (1:1000, Proteintech, China), rabbit anti‐Adam10 (1:1000, Selleck, USA), rabbit anti‐Vimentin (1:1000, Selleck, USA), mouse anti‐Gapdh (1:10000, Proteintech, China) and mouse anti‐beta tubulin(1:10000, Proteintech, China). After TBST washes, membranes were incubated 1 h with HRP‐conjugated secondaries including goat anti‐mouse (1:10000, Proteintech, China) and goat anti‐rabbit (1:10000; Proteintech, China). At last, signal detection employed ECL Western blotting kit (Beyotime, China) and chemiluminescence imaging (Bio Rad, USA). Band intensity quantification used ImageJ software (NIH, USA).

### 4.13 Immunofluorescence Staining

Rats were transcardially perfused with ice‐cold phosphate‐buffered saline followed by 4% paraformaldehyde (Biosharp, China). The brains were post‐fixed in the same fixative for 24 h at 4°C, cryoprotected in 30% sucrose until sinking, and subsequently sectioned coronally into 14‐µm thick slices. For staining, brain sections underwent antigen retrieval in citrate buffer (95°C, 15 min), followed by blocking with 5% goat serum (Servicebio, China) in PBST (0.3% Triton X‐100) for 1 h at room temperature. The sections were then incubated overnight at 4°C with the following primary antibodies: rabbit anti‐CCR2 (1:100, Thermo Fisher Scientific, USA), rabbit anti‐GAS6 (1:100, Proteintech, China), guinea pig anti‐NeuN (1:500; Oasisbiofarm, China), and mouse anti‐CD31 (1:100, abcam, USA). After washing, the sections were incubated for 2 h at room temperature with corresponding secondary antibodies including Goat‐anti‐rabbit IgG, AF488 (1:1000, Oasisbiofarm, China), Goat‐anti‐mouse IgG, AF594 (1:1000, Oasisbiofarm, China), Goat‐anti‐rabbit IgG, AF594 (1:1000, Oasisbiofarm, China), Goat‐anti‐guinea pig IgG, AF488 (1:1000; Oasisbiofarm, China). Terminal dUTP Nick End Labeling (TUNEL) was detected as followed methods: washing sections three times with Phosphate Buffered Saline (PBS) before applying the TUNEL mixture from the TUNEL detection kit (Beyotime, China). Cell nuclei were counterstained with DAPI (Servicebio, China). Images were obtained by fluorescence microscope (Nikon ECLIPSE Ci, Japan).

### 4.14 Transmission Electron Microscopy

Periventricular tissues (approximately 1 mm^3^) were dissected and immersion‐fixed in ice‐cold 2.5% glutaraldehyde/0.1M phosphate buffer (pH 7.4) for 24 h at 4°C. Samples were rinsed 4× with 0.1M phosphate buffer (15min/rinse), then post‐fixed in 1% osmium tetroxide/0.1M phosphate buffer (pH 7.4) for 2 h at 4°C. After quadruple distilled water rinses, specimens underwent graded ethanol concentrations: 50% (15min), 70% (15min), 90% (15min), 95% (15min), and 100% (2 ×10min). Tissues were transitioned through propylene oxide (2 × 15min) and embedded in EPON 812 resin via graded resin: propylene oxide mixtures (1:2, 1:1, 2:1; 2 h each). Polymerization occurred at 60°C for 48 h. Ultrathin sections (60–90 nm) collected on 200‐mesh copper grids were stained with 2% uranyl acetate (15min, dark) and with Reynold's lead (5min). Sections were imaged using a transmission electron microscope (JEOL JEM‐1400Flash, JEOL Ltd., Japan) at 80 kV accelerating voltage to observe the BBB ultrastructure.

### 4.15 Statistical Analysis

Data normality was verified using the Shapiro‐Wilk test. Parametric analyses were performed as follows: (1) Two‐group comparisons: Unpaired Student's *t*‐test. (2) Three or more groups’ comparisons: One‐way ANOVA with Tukey's post hoc test. (3) Factorial designs: Two‐way ANOVA with Šidák's multiple comparisons. Continuous data is presented as mean ± standard deviation (SD) unless otherwise specified in figure legends. All analyses incorporated ≥ 3 biological replicates per condition. For correlation analyses between CSF leukocyte counts and clinical scores (Hunt‑Hess and mRS), Spearman's rank correlation coefficient was used due to the non‑normal distribution of the clinical scoring data. Given the exploratory nature of this analysis and the limited number of correlation tests performed, no multiple testing correction was applied. Statistical significance was defined as p ≤ 0.05. Analyses were conducted in GraphPad Prism 9.15 with visualization of results.

## Author Contributions

**Yajun Zhu**: funding acquisition, Writing – original draft, conceptualization, methodology, data curation, software, investigation. **Zichao Huang**: methodology, software, data curation, conceptualization, validation. **Xi Rong**: conceptualization, methodology, software, data curation, formal analysis. **Xingwei Lei**: conceptualization, methodology, software, data curation, investigation. **Fuming Liang**: methodology. **Xiaoguo Li**: methodology. **Jiru Zhou**: methodology. **Yu Wang**: visualization. **Yifan Huang**: visualization. **Shuang Tang**: resources, funding acquisition. **Jianjun Zhong**: supervision, resources. **Jin Yan**: methodology, supervision. **Hongji Deng**: writing – review and editing, project administration, supervision. **Chun Zeng**: writing – review and editing, project administration, supervision, resources. **Zongduo Guo**: writing – review and editing, project administration, resources, funding acquisition, supervision.

## Conflicts of Interest

The authors declare no conflicts of interest.

## Supporting information




**Supporting File**: advs77946‐sup‐0001‐SuppMat.docx.

## Data Availability

The data that support the findings of this study are available from the corresponding author upon reasonable request.
