# Supplementary material for: Remote Ischemic Post‐Conditioning Preserves the Neurovascular Unit by Brain‐Derived Gas6 That Drives Monocyte‐Derived Macrophage Reprogramming via Axl and Vimentin after SAH
Source: Adv Sci (Weinh). 2026 Sep 27:e77946. Online ahead of print. doi: 10.1002/advs.77946 (PMC13616258; doi:10.1002/advs.77946)
Supplement: Supplementary file 1 [file ADVS-9999-e77946-s001.docx]

**Remote Ischemic Post-conditioning Preserves the Neurovascular Unit by Brain-derived Gas6 that Drives Monocyte-derived Macrophage Reprogramming via Axl and Vimentin after SAH**

*Yajun Zhu**^#1^, Zichao Huang**^#1^,* *Xi Rong^#3^, Xingwei Lei**^#1^, Fuming Liang^1^, Xiaoguo Li^1^, Jiru Zhou**^1^, Yu Wang^1^, Yifan Huang^1^, Shuang Tang^2^, Jianjun Zhong^1^, Jin Yan^1^, Hongji Deng^1*^, Chun Zeng^*2^, Zongduo Guo^*1^*

Yajun Zhu, Zichao Huang, Xingwei Lei, Fuming Liang, Xiaoguo Li, Jiru Zhou, Yu Wang, Yifan Huang, Jianjun Zhong, Jin Yan, Hongji Deng, Zongduo Guo

^1^Department of Neurosurgery, The First Affiliated Hospital of Chongqing Medical University, Chongqing, China. 400016

***Correspondence:**

Zongduo Guo, MD

Email: stonegzd@163.com

Hongji Deng, MD

Email: hengistdeng@163.com

Shuang Tang, Chun Zeng

^2^Department of Cerebrovascular Diseases, Suining Central Hospital, Sichuan, China. 629000

***Correspondence:**

Chun Zeng, MD

Email: cengchun@sns120.cn

Xi Rong

^3^West China Hospital, Sichuan University, Sichuan, China. 610041

**Content of Supplementary**

1. Figure S1. Time‑dependent infiltration of CCR2⁺ cells into the periventricular region after SAH.

2. Figure S2. Single‑cell RNA sequencing reveals cell type distribution changes after SAH and RIPostC.

3. Figure S3. RIPostC reduces neuronal apoptosis after SAH.

4. Figure S4. Dose‑dependent effect of Withaferin A (WFA) on Vimentin protein expression after SAH.

5. Figure S5. Immunofluorescence double staining for CD31 (red) and Gas6 (green) in the periventricular region of SAH rats.

6. Figure S6. Validation of adenovirus mediated Gas6 knockdown in the rat brain.

7. Table S1. Clinical baseline characteristics based on patient disease severity (Hunt-Hess score).

8. Table S2. Clinical baseline characteristics based on patient prognosis (mRS).

9. Other supplementary: Uncropped Western blot membranes with molecular weight markers for all protein detections.

**Figure S1. Time‑dependent infiltration of CCR2⁺ cells into the periventricular region after SAH.**

Representative immunofluorescence images (left) and semi-quantitative analysis (right) of CCR2⁺ cells (green) in the periventricular region at 1, 3, and 7 days after SAH. Nuclei were counterstained with DAPI (blue). Scale bar, 500 μm. n = 6 per time point. Data are presented as mean ± SD. *p < 0.05, **p < 0.01, ***p < 0.001, ****p < 0.0001, ns: not significant.

**Figure S2. Single‑cell RNA sequencing reveals cell type distribution changes after SAH and RIPostC.**

Data are presented as mean ± SD. *p < 0.05, **p < 0.01, ***p < 0.001, ****p < 0.0001, ns: not significant.

**Figure S3. RIPostC reduces neuronal apoptosis after SAH.**

Representative immunofluorescence images (left) of NeuN (red), TUNEL (green), and DAPI (blue) in the periventricular region of Sham, SAH, and SAH + RIPostC groups. White arrowheads indicate NeuN⁺TUNEL⁺ double‑positive neurons. Semi-quantitative analysis (right). Scale bar, 100 and 200 μm. n = 6 per group.

**Figure S4. Dose‑dependent effect of Withaferin A (WFA) on Vimentin protein expression after SAH.**

Western blot analysis of Vimentin expression in periventricular tissues from SAH rats treated with vehicle or WFA at 1, 2, or 4 mg/kg. Representative blots for Vimentin (left). Quantitative analysis of Vimentin protein levels (right). n = 6 per group.

**Figure S5. Immunofluorescence double staining for CD31 (red) and Gas6 (green) in the periventricular region of SAH rats. Nuclei were counterstained with DAPI (blue). Negligible colocalization was observed. Scale bar, 10 and100 μm.**

**Figure S6. Validation of Adeno-Associated Virus-Mediated Gas6 Knockdown in the rat brain.**

(A) Representative immunofluorescence image showing EGFP (green) and NeuN (red) expression as a marker of successful Adeno-Associated Virus infection in the periventricular region. Scale bar, 200 μm. (B) Western blot validation of Gas6 expression in rats injected with sh‑Gas6 adenovirus. n = 3 per group. Data are presented as mean ± SD. *p < 0.05, **p < 0.01, ***p < 0.001, ****p < 0.0001, ns: not significant.

| **Table S1. Clinical baseline characteristics based on patient disease severity (Hunt-Hess score)** | | | | |
| --- | --- | --- | --- | --- |
| Variables | Severe patients (Hunt Hess ≥ 3,  n = 84) | Mild patients (Hunt Hess ＜ 3, n = 321) | t/χ² | p |
| Age (year) | 57.46±12.13 | 58.34±10.95 | -0.64 | 0.52 |
| Gender |  |  | 0.02 | 0.89 |
| Male | 33(39.29) | 121(37.69) |  |  |
| Female | 51(60.71) | 200(62.31) |  |  |
| Hypertension | 50(59.52) | 151(47.04) | 3.67 | 0.06 |
| Diabetes | 7(8.33) | 13(4.05) | 1.77 | 0.18 |
| Hyperlipidemia | 6(7.14) | 17(5.30) | 0.15 | 0.70 |
| Ischemia | 3(3.57) | 25(7.79) | 1.24 | 0.27 |
| Coronary heart disease (CHD) | 1(1.19) | 8(2.49) | 0.09 | 0.76 |
| Current smoker | 9(10.71) | 32(9.97) | 0.00 | 1.00 |
| Alcohol consumption | 7(8.33) | 15(4.67) | 1.10 | 0.30 |
| * p<0.05 ** p<0.01 | | | | |

| **Table S2. Clinical baseline characteristics based on patient prognosis (mRS)** | | | | |
| --- | --- | --- | --- | --- |
| Variables | Adverse prognosis (mRS ≥ 3, n = 60) | Good prognosis  ( mRS ＜ 3, n = 345) | t/χ² | p |
| Age (year) | 60.72±12.07 | 57.71±11.00 | 1.92 | 0.06 |
| Gender |  |  | 0.14 | 0.71 |
| Male | 21(35.00) | 133(38.55) |  |  |
| Female | 39(65.00) | 212(61.45) |  |  |
| Hypertension | 43(71.67) | 158(45.80) | 12.67 | 0.001** |
| Diabetes | 4(6.67) | 16(4.64) | 0.12 | 0.73 |
| Hyperlipidemia | 6(10.00) | 17(4.93) | 1.60 | 0.21 |
| Ischemia | 9(15.00) | 19(5.51) | 5.76 | 0.02* |
| Coronary heart disease (CHD) | 1(1.67) | 8(2.32) | 0.00 | 1.00 |
| Current smoker | 5(8.33) | 36(10.43) | 0.07 | 0.79 |
| Alcohol consumption | 5(8.33) | 17(4.93) | 0.59 | 0.44 |
| * p<0.05 ** p<0.01 | | | | |

**Other supplementary:** Uncropped Western blot membranes with molecular weight markers for all protein detections.

**(a**) Uncropped membrane for Adam10 with marker, and the same membrane was reprobed for GAPDH (corresponding to Fig. 3E).

**(b)** Uncropped membrane for Vimentin with marker, and the same membrane was reprobed for GAPDH (corresponding to Fig. 3E).

**(c)** Uncropped membrane for Axl with marker, and a separate membrane for GAPDH is shown, specify (corresponding to Fig. 4H).

**(d)** Uncropped membrane for Mertk with marker, and a separate membrane for GAPDH is shown, specify (corresponding to Fig. 4H).

**(e)** Uncropped membrane for Gas6 with marker, and a separate membrane for GAPDH is shown, specify (corresponding to Fig. 4H).

**(f)** Uncropped membrane for Vimentin with marker, and the same membrane was reprobed for GAPDH (corresponding to Fig. 7B).

**(g)** Uncropped membrane for Vimentin with marker, and the same membrane was reprobed for GAPDH (corresponding to Fig. S4).

**(h)** Uncropped membrane for Gas6 with marker, and the same membrane was reprobed for β-tubulin (corresponding to Fig. S6B).
